# ARGLU1 is a transcriptional coactivator and splicing regulator important for stress hormone signaling and development

**DOI:** 10.1093/nar/gkz010

**Published:** 2019-01-30

**Authors:** Lilia Magomedova, Jens Tiefenbach, Emma Zilberman, Florian Le Billan, Veronique Voisin, Michael Saikali, Vincent Boivin, Melanie Robitaille, Serge Gueroussov, Manuel Irimia, Debashish Ray, Rucha Patel, ChangJiang Xu, Pancharatnam Jeyasuria, Gary D Bader, Timothy R Hughes, Quaid D Morris, Michelle S Scott, Henry Krause, Stephane Angers, Benjamin J Blencowe, Carolyn L Cummins

**Affiliations:** 1 Department of Pharmaceutical Sciences, University of Toronto, Toronto, ON M5S 3M2, Canada; 2 Donnelly Centre for Cellular and Biomolecular Research, University of Toronto, Toronto, ON M5S 3E1, Canada; 3 Département de biochimie, Faculté de médecine et des sciences de la santé, Université de Sherbrooke, Sherbrooke, QC J1E 4K8, Canada; 4 Department of Molecular Genetics, University of Toronto, Toronto, ON M5S 1A8, Canada; 5 Department of Obstetrics and Gynecology, Wayne State University Perinatal Initiative, School of Medicine, Wayne State University, Detroit, MI, USA; 6 Department of Biochemistry,University of Toronto, Toronto, ON M5S 1A8, Canada

## Abstract

Stress hormones bind and activate the glucocorticoid receptor (GR) in many tissues including the brain. We identified arginine and glutamate rich 1 (ARGLU1) in a screen for new modulators of glucocorticoid signaling in the CNS. Biochemical studies show that the glutamate rich C-terminus of ARGLU1 coactivates multiple nuclear receptors including the glucocorticoid receptor (GR) and the arginine rich N-terminus interacts with splicing factors and binds to RNA. RNA-seq of neural cells depleted of ARGLU1 revealed significant changes in the expression and alternative splicing of distinct genes involved in neurogenesis. Loss of ARGLU1 is embryonic lethal in mice, and knockdown in zebrafish causes neurodevelopmental and heart defects. Treatment with dexamethasone, a GR activator, also induces changes in the pattern of alternatively spliced genes, many of which were lost when ARGLU1 was absent. Importantly, the genes found to be alternatively spliced in response to glucocorticoid treatment were distinct from those under transcriptional control by GR, suggesting an additional mechanism of glucocorticoid action is present in neural cells. Our results thus show that ARGLU1 is a novel factor for embryonic development that modulates basal transcription and alternative splicing in neural cells with consequences for glucocorticoid signaling.

## INTRODUCTION

The glucocorticoid receptor (GR) plays a fundamental role in coordinating the transcriptional response to stress hormones, such as cortisol, and is essential for organismal development, glucose homeostasis and immune function (1). In the brain, GR is highly expressed in the hippocampus where it has been shown to play a central role in modulating the proliferation and differentiation of neural stem and progenitor cells (2–4).

GR and other members of the nuclear receptor superfamily share highly conserved domains including the zinc-finger DNA-binding domain (DBD) and a carboxy terminal ligand-binding domain (LBD) attached to a ligand dependent activation function domain (AF2). In the absence of ligand, GR is complexed to chaperone proteins in the cytosol that dissociate upon ligand binding and unmask a nuclear localization signal. GR then translocates to the nucleus where it regulates gene expression by binding to GR response DNA elements or to other transcription factors.

Ligand-bound GR is known to interact with members of the p160 coactivator family including steroid receptor coactivator 1 (SRC1) and glucocorticoid receptor interacting protein GRIP1 (TIF2). SRC1 and TIF2 can recruit histone acetyltransferase enzymes, resulting in changes in chromatin structure (5–7). Interactions between coactivators and other transcription factors and regulatory proteins help to recruit RNA polymerase II and initiate gene transcription (8). Coregulators play roles in every step of transcription including chromatin remodeling, initiation, elongation, and termination (9). Some coregulators have also been implicated in the regulation of RNA splicing (10–17). While numerous GR coregulators have been identified, their effects on stress-induced corticosteroid signaling in the brain remain largely unexplored. To identify new biological mediators of GR function in the central nervous system (CNS), we performed a high-throughput expression cloning screen examining GR transcriptional activity in response to stress hormones.

Herein, we report that arginine and glutamate rich 1 (ARGLU1) is a highly evolutionarily conserved transcriptional coactivator and RNA splicing modulator. We show in neural cells that glucocorticoid signaling, through dexamethasone (Dex) treatment, not only affects transcription but also changes the alternative splicing landscape of genes important for chromatin organization and neuronal differentiation, some of which are also ARGLU1-dependent. These functions were previously unknown as ARGLU1 had only been shown to interact with the mediator complex MED1 and affect estrogen receptor signaling (18). We show that ARGLU1 is highly expressed in the CNS, and loss of ARGLU1 is embryonic lethal in mice. Our data support a model in which ARGLU1 uses its distinct domains to control mRNA on two levels: by changing gene expression and alternative splicing in pathways such as histone chromatin organization and neurogenesis.

## MATERIALS AND METHODS

For procedures listed below, additional details are available in the Supplemental Experimental Procedures.

### High-throughput expression screen and transfection assays

Electromax™ DH10B™ *Escherichia coli* cells (Invitrogen, Carlsbad, CA, USA) were transformed with 10 ng of normalized human brain cDNA library, diluted and grown overnight in deep 96-well plates (Sigma, St. Louis, MO, USA). The following morning, plasmid DNA was isolated from the bacterial cultures using the GenElute™ HP 96 well plasmid midiprep kit (Sigma). Screening was performed by transfecting pools of the extracted plasmid DNA into HEK293 cells using calcium phosphate in the presence of indicated controls (19). Six hours post-transfection, cells were treated with vehicle (ethanol) or 300 nM cortisol. Cells were harvested 14–16 h later for luciferase and β-galactosidase activity, as previously described (19). Neuro-2a (N2a) cells were transfected in suspension with siRNA against *Arglu1* (D-057082-02; 5′-GCCAAACGCAUCAUGGAAA-3′) or with the non-targeting Control siRNA (siGENOME Non-Targeting siRNA Pool #2; D-001206-14-05) using RNAiMax as per manufacturer's instructions.

### Confocal microscopy

HEK293 or Neuro-2a cells were grown on a poly-d-lysine coated cover slips and co-transfected with mCherry-hARGLU1 and GFP-hGR, GFP-mSRSF2, GFP-hPUF60 or GFP-hU2AF2. After 48 h, cells were treated with either vehicle (ethanol) or 100 nM Dex for 4 h and then fixed using 4% PFA (pH 7.4). Vectashield (Vector laboratories, Burlingame, CA, USA) mounting media with DAPI was used to mount the coverslips on glass slides and visualized as described in supplemental experimental procedures.

### RNA isolation, cDNA synthesis, and real-time qPCR analysis

Total RNA was extracted with RNA STAT-60 (Tel-Test Inc, Friendswood, TX, USA), treated with DNase I (Roche, Laval, QC, Canada) and reverse transcribed into cDNA with random hexamer primers using the High Capacity Reverse Transcription System (ABI, Burlington, ON, Canada) as previously described (20). Real-time quantitative PCR reactions for gene expression analysis were performed on an ABI 7900HT Fast Real-Time PCR System in a 384-well plate format according to Bookout *et al.* (20). Primers were designed to span exon-exon junctions and have an amplicon size of 50–150 bp using Primer Express Software v3.0. Primers were then validated in house and had a slope of −3.33 ± 0.15, with *R*^2^ value of above 0.98 and a single peak in the dissociation curve. Relative mRNA levels were calculated using the comparative Ct method (20). For the tissue library mRNA analysis, the efficiency-corrected ΔCt method was used.

### Proteomics assays

Protein-interaction complexes from HEK293 cells stably expressing BirA*tagged hARGLU1 or tagged-hGR were extracted and subjected to mass spectrometry analysis as described in supplemental methods. Rapid Immunoprecipitation Mass spectrometry of Endogenous proteins (RIME) was performed in N2a cells as previously described (21) using 8 μg of normal rabbit IgG (Santa Cruz, sc-2027), anti-ARGLU1 (Novus Biologicals, NBP1-87921) or anti-GR antibody (Santa Cruz, sc-1004).

### Co-immunoprecipitation assays and Western blot analysis

HEK293 cells were grown in 100 mm plates and transiently transfected with 5 μg of HA-hARGLU1 together with 5 μg of either FLAG-hPUF60, FLAG-hU2AF2 or FLAG-hJMJD6. Forty-eight hours later, cells were harvested and protein was extracted. Protein lysates were pre-cleared with protein G beads, split into three tubes and incubated with 5 μg of anti-FLAG (1:1000; Sigma, F1804) or anti-HA (1:1000; Cell Signaling, 3724S) antibody. The next day, 50 μl of protein G agarose was added to the lysates and incubated on a rotator for 3–4 h at 4°C. Proteins were eluted in lithium dodecyl sulfate buffer (LDS, Invitrogen) and resolved on a 4–20% gradient SDS gel (Bio-Rad, Hercules, CA, USA).

### RNA-seq studies and analysis

N2a cells were transfected with siGENOME siRNA (Dharmacon) using RNAiMAX reagent (Invitrogen). mRNA was pooled by treatment group (*n* = 3/group) and mRNA enriched Illumina TruSeq V2 RNA libraries were prepared. Samples were sequenced at the Donnelly Sequencing Centre (University of Toronto) on Illumina HiSeq2500. Transcriptome-wide alternative splicing and gene expression profiling were performed using a previously described workflow (22).

### Chromatin Immunoprecipitation and genomic qPCR

N2a cells were transfected with siArglu1 or with the non-targeting siControl and were grown in 10 cm plates. At 90% confluency (8 million cells/plate), N2a cells were treated 1h with 100 nM Dex or vehicle (1:1000 ethanol). After formaldehyde fixation, chromatin was extracted and sheared, as described in the supplemental experimental procedures. Chromatin was then immunoprecipitated with anti-GR (Santa Cruz, sc-393232x), anti-ARGLU1 (Novus Biologicals, NBP1-87921) or mouse control IgG antibody (Santa Cruz, sc-2025) preincubated with protein G coated magnetic beads. The beads were washed, and immunoprecipitated DNA was eluted, de-crosslinked and purified. Purified DNA was quantified by genomic qPCR using serial dilutions of the input as a standard curve, performed in triplicates. Results are expressed as the percent enrichment of bound DNA compared to each input.

### RNAcompete studies

The procedure was performed as previously described (23). Briefly, full-length or truncated GST-tagged ARGLU1 proteins were purified from bacteria (23). Individual proteins (20 pmol) and RNA pool (1.5 nmol) were incubated in 1 mL of binding buffer (20 mM HEPES pH 7.8, 80 mM KCl, 20 mM NaCl, 10% glycerol, 2 mM DTT, 0.1 mg/ml BSA) containing 20 μl of glutathione sepharose 4B beads (GE Healthcare, pre-washed three times in binding buffer) for 30 min at 4°C. Beads were then washed and the recovered RNA was directly labeled with either Cy3 or Cy5 and subsequently hybridized to the Agilent 244K microarray used to generate the RNA pool. One-sided Z-scores were calculated for the motifs as previously described (23).

### Animal experiments

Three targeted ES cell lines heterozygous for the knockout-first-reporter tagged *Arglu1^tm1a(EUCOMM)Hmgu^* allele were obtained from European Conditional Mouse Mutagenesis Program (EUCOMM). The generation and breeding of the transgenic animals is described in the extended experimental procedures. C57Bl/6 mice used for tissue collection were housed in a temperature and light-controlled environment and fed *ad libitum* the 2016S diet (Harlan Teklad Mississauga, ON, Canada). Zebrafish were maintained at 28.5°C on a 14/10 h light/dark cycle and staged relative to hours post-fertilization. All animal studies were performed according to the recommendations of the Faculty of Medicine and Pharmacy Animal Care Committee at the University of Toronto (Toronto, ON, Canada).

### Zebrafish morpholino injections and *in situ* hybridization

Translation-blocking morpholinos (MO) targeting zebrafish *arglu1a, arglu1b* and *p53* genes were purchased from Gene Tools, LLC (Philomath, OR, USA). Morpholinos were injected alone or in combination with the indicated rescue RNA into one-cell stage embryos using standard techniques. Whole mount *in situ* hybridization was carried out as previously described with the indicated antisense RNA probes (24). Rescue RNA and antisense RNA probes were synthesized using mMESSAGE mMACHINE (Ambion, Austin, TX, USA). Embryos were imaged at indicated times after fertilization.

### Statistical analysis

Data are presented as mean ± SD unless otherwise indicated. One-way ANOVA followed by the Newman–Keuls test was used to compare more than two groups. For comparison between two groups the unpaired Student's t-test was performed except for the comparison of the fold changes in Supplementary Table S1 where the Wilcoxon matched pairs test was used. All tests were performed using GraphPad Prism6. *P* < 0.05 was considered significant.

## RESULTS

### ARGLU1 is identified as a transcriptional coactivator of GR from a cDNA expression cloning screen

**Figure 1.**
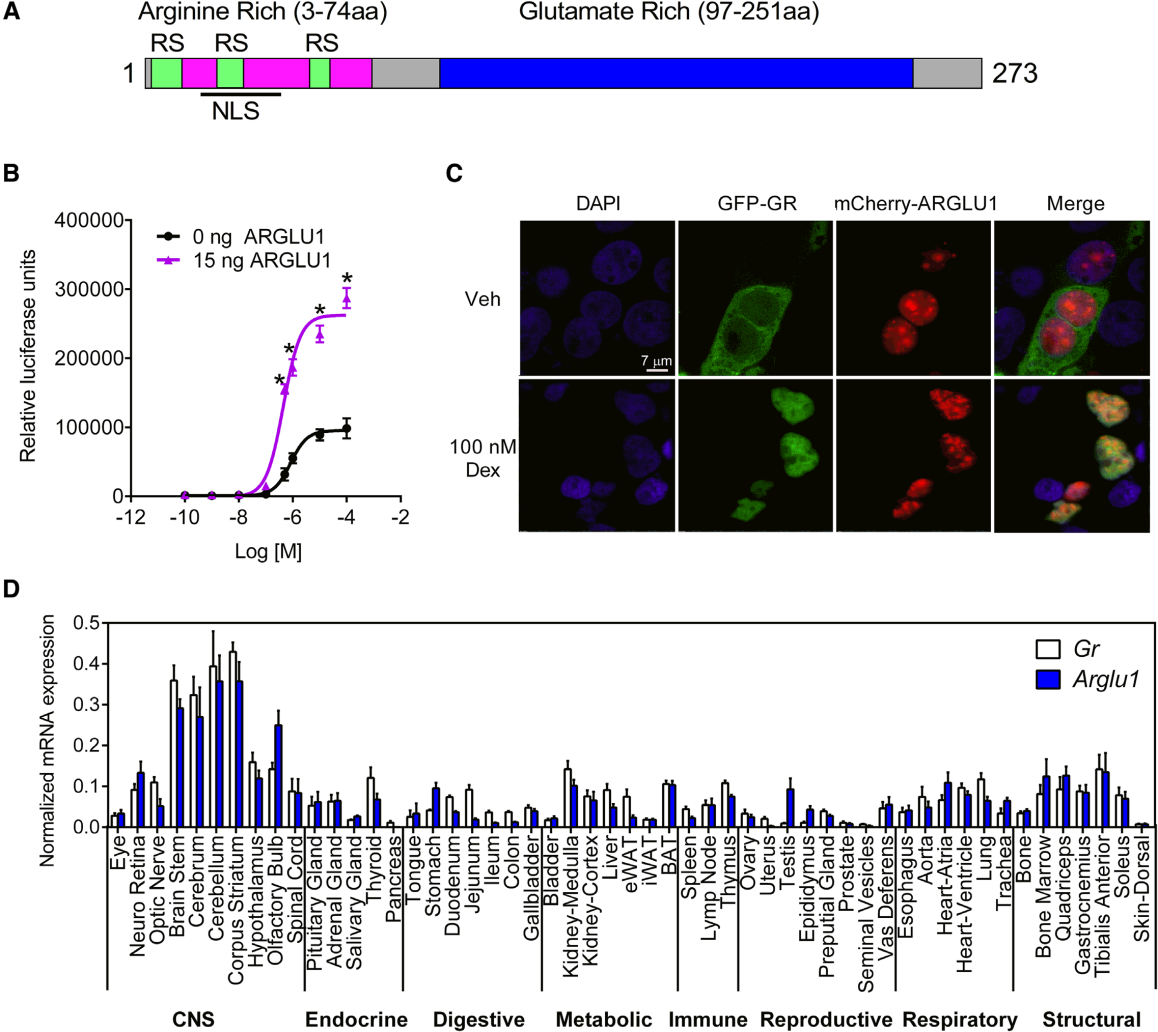
ARGLU1 is a GR transcriptional coactivator. (**A**) Schematic of ARGLU1 domains. NLS, nuclear localization sequence; RS, arginine-serine repeats. (**B**) HEK293 cells transfected with the GAL4-GR/UAS-luc reporter with a constant amount of CMX-ARGLU1 (15 ng/well) and increasing concentrations of cortisol. Data represent the mean ± SD. Student's *t*-test *P < 0.05 versus 0 ng ARGLU1. (**C**) GFP-GR (green) and mCherry-ARGLU1 (red) were co-transfected into HEK293 cells and treated with vehicle (EtOH) or 100 nM Dex for 4 h. DAPI (blue) was used to stain the nuclei. The Pearson's correlation coefficient for Veh is −0.03 ± 0.05 and for Dex is 0.52 ± 0.07. (**D**) Tissues from male C57Bl/6 mice 4 months of age were collected. Ovary and uterus were from female mice. RNA was extracted and samples were DNase treated, reverse transcribed, and analyzed by qPCR (efficiency-corrected ΔCt method). *36b4* was used as an endogenous reference RNA normalizer gene. Data represent the mean ± SD of individual qPCR well. Each tissue was pooled from at least 2 animals. eWAT, epididymal white adipose tissue; iWAT, inguinal white adipose tissue; BAT, brown adipose tissue. See also Supplementary Figures S1–S3.

In an effort to identify novel proteins influencing stress hormone signaling, an expression cloning assay measuring GR activity was optimized in HEK293 cells and screened in the presence of cDNA pools from a normalized human brain cDNA library. The GC-responsive reporter system consisted of a plasmid encoding the fusion protein of human GR ligand binding domain linked to the yeast GAL4 DNA binding domain (GAL4-GR), and a luciferase reporter plasmid driven by four GAL4 upstream activating sequences (UAS-luc). Cortisol, an endogenous GR ligand and stress hormone, was used at a sub-saturating dose of 300 nM. Hits were considered positive if they yielded a luciferase signal >2-fold compared to control transfected cells. Note that because only the C-terminal ligand binding domain was present in our GAL4-GR construct, our screen would not recover proteins that interacted via the N-terminal AF1 domain. A 2-fold increase or decrease in luciferase activity with co-transfection of the known GR coactivator, TIF2, and corepressor, RIP140, respectively, was detectable when diluted 50-fold with an empty plasmid, indicating that a cDNA pool size of ∼50 could be effectively screened in our assay. Subsequent rounds of sib selection were performed screening at 12 clones/well and 1 clone/well (Supplementary Figure S1A and B). Following up one of the hits led to the identification of a cDNA encoding the arginine and glutamate rich 1 (ARGLU1) protein (Figure 1A).

To confirm that ARGLU1 was a GR coactivator, we transfected HEK293 cells with increasing amounts of ARGLU1 and observed a dose-dependent increase in GR transcriptional activity (Supplementary Figure S1C). Likewise, we found that ARGLU1 significantly increased the maximal activation of GAL4-GR when tested in a dose response assay with cortisol, suggesting that ARGLU1 behaves analogously to other coactivators for this receptor (Figure 1B).

### ARGLU1 is a highly evolutionarily conserved nuclear protein that is ubiquitously expressed

ARGLU1 is a 273 amino acid protein (33 kDa) named because of its two distinct regions. The N-terminus is rich in positively charged arginine amino acids and the C-terminus is composed of glutamate rich amino acids (Figure 1A and Supplementary Figure S2A). The evolutionary conservation between orthologs of ARGLU1 is very high with 99% sequence identity between mouse and human ARGLU1 (Supplementary Figure S2A). Functional complementation was shown for mouse and zebrafish ARGLU1 when tested in a human GR co-transfection assay (Supplementary Figure S2B). ARGLU1 is not related to other known NR coregulators and is known to interact with MED1 and influence estrogen receptor-regulated transcription (18). A bipartite nuclear localization sequence was found near the N-terminal end along with several arginine and serine (RS)-rich repeats (Supplementary Figure S2A). Interestingly, proteins which contain arginine and serine dipeptide repeats, such as those in the SR and SR-related families, play important roles in constitutive and alternative splicing by participating in spliceosome formation and splice site selection (25). While no formal RNA recognition motifs are present in ARGLU1, it can be classified as an ‘SR-related’ protein (26–28). In the glutamate-rich domain we located two non-classical LXXLL motifs, LLXXL (172–176 aa) and LXXIL (201–205 aa) (Supplementary Figure S2A). LXXL/IL motifs are canonical interaction sequences found in many NRs coactivators that facilitate binding to the AF2 domains of the receptors.

We next examined the intracellular distribution of ARGLU1 and compared it to that of GR in the presence and absence of the GC, Dex, in HEK293 cells. As expected, GR was localized primarily in the cytoplasm but translocated into the nucleus when Dex was added (Figure 1C). ARGLU1 was constitutively nuclear and found both in the nucleoplasm and in a distinct punctate pattern. Studies performed with GFP-tagged nuclear speckle marker SRSF2 demonstrated very high co-localization with ARGLU1 with a Pearson correlation coefficient (PCC) of 0.83 ± 0.05 (Supplementary Figure S3), suggesting this punctate pattern corresponds to nuclear speckles. While we observed a statistically significant overlap between the localization of nuclear GR (+Dex) and ARGLU1 [PCC of 0.52 ± 0.07], this was not striking, suggesting that the bulk of the proteins do not overlap and no structures are defined by the overlap in signal that does occur.

To determine the tissue distribution of *Arglu1*, we measured its mRNA expression in 53 different C57Bl/6 mouse tissues by RT-qPCR. *Arglu1* is ubiquitously expressed with the highest level of expression in the central nervous system. We compared the tissue distribution of *Gr* (*Nr3c1*) to that of *Arglu1* and found them highly correlated with only a few exceptions (i.e., *Arglu1* is low in uterus and pancreas; *Gr* is low in testis, Figure 1D).

### ARGLU1 is a general NR coactivator that acts in concert with other coactivators via its C-terminal domain

To test whether ARGLU1 can coactivate other members of the NR superfamily, we used the GAL4-NR-LBD/UAS-luciferase system. ARGLU1 increased the ligand-induced transcriptional activity of several steroid receptors (GR, ERα, MR, PR, ERβ) as well as a number of RXR heterodimer partners (PPARα,β,γ, LXRα,β and VDR), while no effect was observed for GAL4-FXR (Supplementary Figure S4). Interestingly, ARGLU1 appeared to have the greatest effect on ligand-dependent coactivation of GAL4-GR and GAL4-ERα.

**Figure 2.**
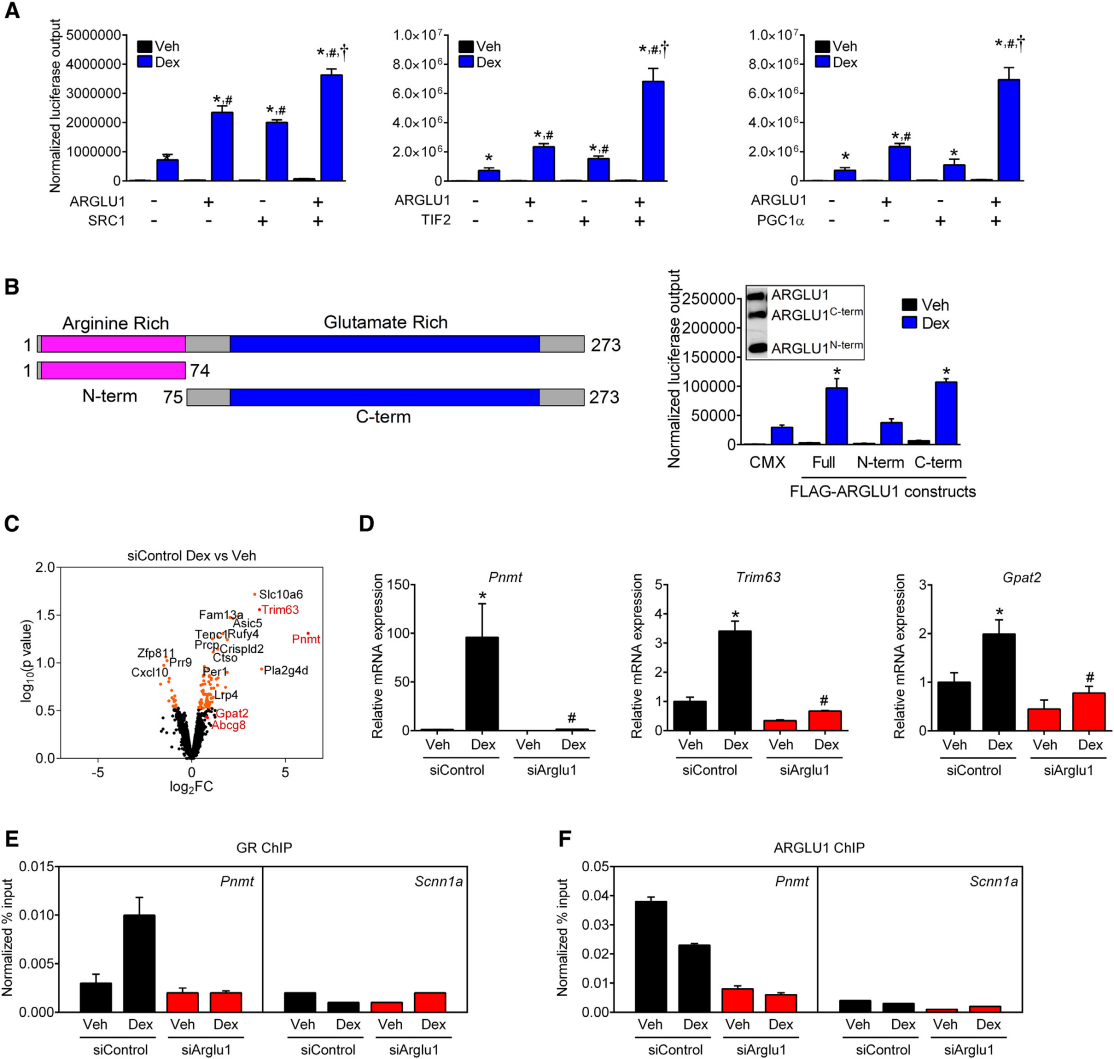
ARGLU1 is required for a full GR transcriptional response. (**A**) HEK293 cells were transiently transfected with CMX-hGR, MMTV-luc and ARGLU1 alone or in combination with known NR coactivators: SRC1, TIF2 and PGC1α; followed by administration of EtOH (Veh) or 100 nM Dex for 16 h. Data represent the mean ± SD (*n* = 3). *P < 0.05 Veh versus Dex for respective condition; ^#^*P* < 0.05 relative to empty vector (CMX)-Dex; ^†^*P* < 0.05 versus ARGLU1-Dex; ANOVA followed by Newman-Keuls test. (**B**) Schematic diagram of ARGLU1 truncations (left) and co-transfection assay of GAL4-GR/UAS-luciferase with 15 ng of ARGLU1 truncations in HEK293 cells treated with EtOH (Veh) or 100 nM Dex (right). CMX was used as a control. Data represent the mean ± SD (*n* = 3). Inset: FLAG-tagged full length ARGLU1 or the indicated truncation mutants were co-expressed in HEK293 cells. *P < 0.05 versus CMX-Dex; ANOVA followed by Newman–Keuls test. (C, D) Neuro-2a cells were transfected with 30 pmol of siControl and siArglu1using RNAiMax for 48 h and then treated with vehicle (EtOH) or 100 nM Dex for 4 h before RNA extraction. (**C**) Volcano plot of log_2_ fold change (log_2_FC) versus p value score (defined in methods from edgeR analysis) for differentially expressed genes. Orange – top 100 genes. Red – genes validated by qPCR. (**D**) Ligand-dependent gene expression changes (Dex vs Veh) following ARGLU1 knockdown. Data represent the mean ± SEM (*n* = 3). *P < 0.05 versus respective Veh, ^#^*P* < 0.05 versus siControl; ANOVA followed by Newman-Keuls test. (E, F) ARGLU1-dependant ligand-induced recruitment of GR on *Pnmt* promoter. N2a cells were transfected with siControl or siArglu1 and treated with vehicle (EtOH) or 100 nM Dex for 1 h before crosslinking and lysis. Chromatin immunoprecipitation was performed on the extracts, using (**E**) anti-GR or (**F**) anti-ARGLU1 antibody. Data represent the mean normalized % input ± SD of one experiment (technical replicates, *n* = 3) repeated at least once. *Scnn1a* exon 2 locus served as negative control for GR recruitment. See also Supplementary Figures S4–S6.

Nuclear receptor coactivators are usually present as part of multi-protein complexes that function together and potentiate each other's activity (29). Here, we examined whether ARGLU1 can act together with other nuclear receptor coactivators to increase GR transcriptional activity. As expected, co-transfecting ARGLU1 in HEK293 cells with full-length hGR and a glucocorticoid responsive promoter linked to a luciferase reporter (MMTV-luc) led to a ligand-dependent increase in luciferase activity (Figure 2A). The magnitude of the ARGLU1 response was similar to that observed when other known nuclear receptor co-regulators such as TIF2, are co-transfected with GR. When co-activators were co-transfected together (i.e. ARGLU1 and TIF2 or ARGLU1 and PGC1α) we observed a synergistic increase in ligand-induced luciferase activity (Figure 2A), implying that ARGLU1 forms a complex with other coactivators and GR to regulate gene expression.

We next determined the molecular domain of ARGLU1 responsible for GR coactivation using co-transfection assays with the GAL4-GR/UAS-luciferase system and truncation mutants of ARGLU1. These studies revealed that the construct containing only the C-terminus (ARGLU1^C-term^) retained the ability to coactivate GR, comparable to that of the full-length protein; whereas, the construct containing only the N-terminus (ARGLU1^N-term^) did not (Figure 2B). Equal protein expression of the truncation mutants was confirmed by western blotting (Figure 2B, inset). To identify whether the LXXL/IL motifs we found in the C-terminal end (Supplementary Figure S2A) were essential for GR coactivation, we mutated the leucine/isoleucine residues to alanine and tested these mutants in the co-transfection assay. We found no significant difference in GR coactivation when either NR-box was mutated or when both were simultaneously mutated (Supplementary Figure S5). These data suggest that while the C-terminal domain is important for activation, these motifs alone cannot account for the ARGLU1 mediated transactivation of GR, thus, other non-canonical interactions are likely involved.

### Regulation of basal and GC-mediated transcription by ARGLU1

In light of the high expression of *Arglu1* in the CNS (Figure 1D), we proceeded to characterize the role of ARGLU1 in mouse neuroblastoma Neuro-2a (N2a) cells, which have been used widely to investigate roles of regulatory factors in neural transcription and AS (28,30). RNA-seq was performed in N2a cells following *Arglu1* knockdown in the presence or absence of Dex. ARGLU1 knockdown resulted in a >90% reduction in mRNA and protein with no effect on GR (*Nr3c1*) expression (Supplementary Figure S6A and B). Next, we examined the gene expression changes in response to Dex in the presence or absence of ARGLU1. We identified significantly differentially expressed Dex-responsive genes using edgeR (*P* < 0.05, Figure 2C and Supplementary Table S1) and confirmed these changes by RT-qPCR (Figure 2D). The fold-change in the expression of 7 differentially expressed genes (Dex/Veh, *P* = 0.0156, Supplementary Table S1) was decreased by the absence of ARGLU1 suggesting that ARGLU1 can potentiate the GC-induced transcriptional changes of these target genes. Interestingly, ARGLU1 knockdown completely abolished Dex-mediated induction of one of the top hits *Pnmt* (Figure 2D). *Pnmt* is involved in epinephrine synthesis and is known to be a direct target of GCs in the adrenal gland (31). Another direct target of GR, *Trim63*, an E3 ubiquitin ligase known to play a role in GC-induced proteolysis, also showed a significant decrease in Dex-mediated induction upon ARGLU1 knockdown (32).

Using chromatin immunoprecipitation in N2a cells, we found that there was recruitment of GR to the glucocorticoid response elements (GRE) of *Pnmt* and *Per1* in response to Dex and that this recruitment was diminished with siARGLU1 knockdown (Figure 2E and Supplementary Figure S6G and H). Furthermore, we found that ARGLU1 is also enriched at the GREs of these target genes even in the absence of Dex and that this recruitment approaches background levels in siARGLU1 N2a cells, as expected (Figure 2F and Supplementary Figure S6H; IgG shown in Supplementary Figure S6I). A locus in *Scnn1a* containing no GRE was used as a negative control. These data suggest that ARGLU1 helps to potentiate strong GR recruitment to these promoters.

When we examined the basal changes between N2a cells treated with siArglu1 versus siControl, we found that 607 genes (out of 12 061 total, *P* < 0.05) were differentially expressed in the absence of a GC ligand (Supplementary Table S1, Supplementary Figure S6J). These changes were validated at a high rate by RT-qPCR and showed a strong correlation with RNA-seq data (*n* = 16 genes tested; *r* = 0.934, Supplementary Figure S6F). Pathway analyses using g:Profiler revealed that ARGLU1 is important basally for heart development, protein localization, chromatin organization and processes involved in neuronal cell function (Supplementary Table S2).

### ARGLU1 interacts with splicing factors at the arginine-rich N-terminal domain and regulates alternative splicing in N2a cells

**Figure 3.**
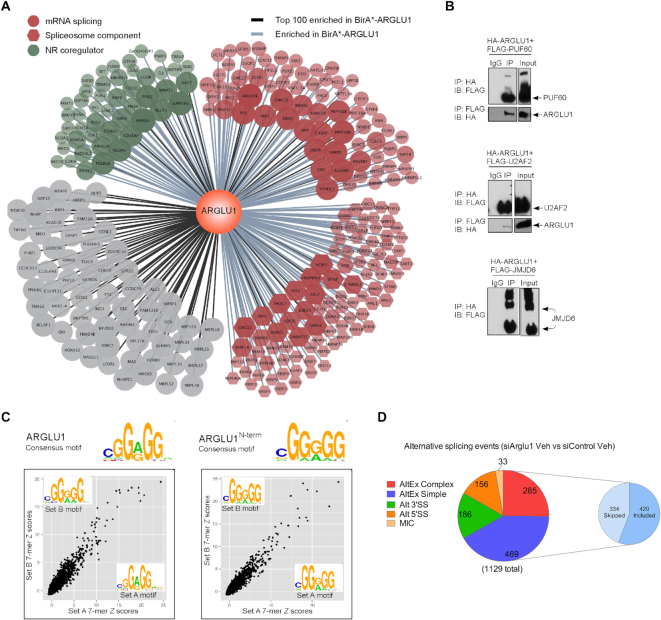
ARGLU1 interacts with splicing factors, binds RNA and modulates the AS response in N2a. (**A**) ARGLU1 protein interaction network using BioID. BirA*-ARGLU1 enriched for 832 proteins (greater than 2-fold vs. BirA* control). The top 100 enriched proteins are connected to the central ARGLU1 node with a black line. Proteins connected with a light blue line are enriched but are not in the top 100. Proteins related to splicing as defined by g:Profiler results were shaded red. The subset of the splicing-related proteins that are specifically linked to the spliceosome (defined by the CORUM database) are represented as red hexagons. Proteins in green are known nuclear receptor coregulators as curated by the Nuclear Receptor Signaling Atlas (www.nursa.org). The size and transparency of the protein nodes are proportional to the protein fold-enrichment. Cytoscape was used to generate the ARGLU1 protein interactome. (**B**) Co-IP of HA-ARGLU1 and selected splicing modulators factors (FLAG-PUF60, FLAG-U2AF2 or FLAG-JMJD6) identified by MS. FLAG-JMJD6 runs as a multimer on a gel. Reverse IPs with the FLAG antibody led to the HA-ARGLU1 being pulled down with FLAG-PUF60 and FLAG-U2AF2. IP, immunoprecipitation; IB, immunoblot. (**C**) RNAcompete results for GST-ARGLU1 and GST-ARGLU1^N-term^ (N-terminus intact). The scatter plots depict correlations between 7-mer Z-scores for set A and set B. Spots corresponding to enriched 7-mers are in the top right corner. Logos for consensus RNA binding motifs, averaged from set A and set B, were generated and shown at the top of the panel (logos for set A and set B are inset). The top ten 7-mers bound by the various ARGLU1 proteins (and corresponding *Z*-scores) are shown in Supplementary Figure S9A. (**D**). Classification of ARGLU1-regulated events. Pie charts showing the distribution of alternative spliced events following ARGLU1 knockdown in N2a cells (in the absence of ligand stimulation). AltEx refers to simple and complex cassette exon events; 3′SS, alternative 3′ splice site; 5′SS, alternative 5′ splice site; MIC, microexon. Pie chart inset: distribution of skipped or included exons within the combined simple and complex cassette exon category with ARGLU1 knockdown. See also Supplementary Figures S7–S9.

To further investigate the mechanism underlying ARGLU1 function we next obtained a more complete picture of its protein interaction landscape. We employed BioID to help detect weakly or transiently interacting proteins that may be missed by standard IP-mass spectrometry (MS). Full length ARGLU1 was fused to a promiscuous *E. coli* biotin protein ligase mutant (BirA R118G, BirA*), which upon introduction into *Flp*-In™ *T-REx*™ 293 cells and addition of biotin results in biotinylation of nearby proteins (33,34). Biotinylated proteins were purified by streptavidin pull down and identified by MS. ARGLU1 was found to interact with many proteins known to be implicated in RNA processing and splicing. Numerous members of the snRNP (small nuclear ribonucleoproteins), hnRNP (heterogeneous nuclear ribonucleoproteins), PRPF (pre-mRNA processing factors), RBM (RNA binding motif proteins), SFRS (splicing factor, arginine/serine-rich) and SF (splicing factors) protein families comprise the ARGLU1 interactome (Figure 3A for summary, Supplementary Table S5 for full list).

The MS results were confirmed with co-IP western blotting experiments in which ARGLU1 was found to interact with PUF60, U2AF2 and JMJD6 (Figure 3B). PUF60 and U2AF2 (also known as U2AF65) bind polypyrimidine tract sequences adjacent to 3′ splice sites and facilitate the recruitment of U2 snRNP during spliceosome assembly, whereas JMJD6 is an enzyme which has been found to hydroxylate U2AF2 and alter its activity (35–37).

In agreement with proteomic data obtained in HEK293 cells, we confirmed that IP-MS of endogenous ARGLU1 protein from N2a cells recovered 150 unique proteins absent from our IgG control (Supplementary Table S6). Using pathway enrichment analysis, we determined that these interacting proteins were enriched for the cellular components consisting of the spliceosomal and RNA polymerase complexes (Supplementary Figure S7), demonstrating consistency between the class of binding partners identified across the two cell systems.

Since ARGLU1 interacts with numerous splicing factors we wanted to determine if it can directly bind specific RNA sequences. To do this, we analyzed full-length GST-ARGLU1 and the two truncated proteins, GST-ARGLU1^N-term^ (N-terminus intact) and GST-ARGLU1^C-term^ (C-terminus intact) using RNAcompete (23,38,39). Purified GST-tagged proteins were incubated with a custom designed RNA pool comprised of ∼240 000 short RNA sequences, 30–41 nucleotides in length. RNAs bound to ARGLU1 were purified, labeled with either Cy3 or Cy5, and hybridized onto custom Agilent 244K microarrays. Computational analysis of the microarray data found full length ARGLU1 preferentially bound to CGG(A/G)GG type k-mers (Figure 3C, Supplementary Figure S9). Interestingly, ARGLU1^N-term^ bound similar G-rich motifs (Figure 3C, Supplementary Figure S9). In contrast, although RNA binding for ARGLU1^C-term^ was observed, no consistent and clear motif was identified (data not shown). Thus, ARGLU1 interacts with a specific RNA motif *via* its N-terminal region.

**Figure 4.**
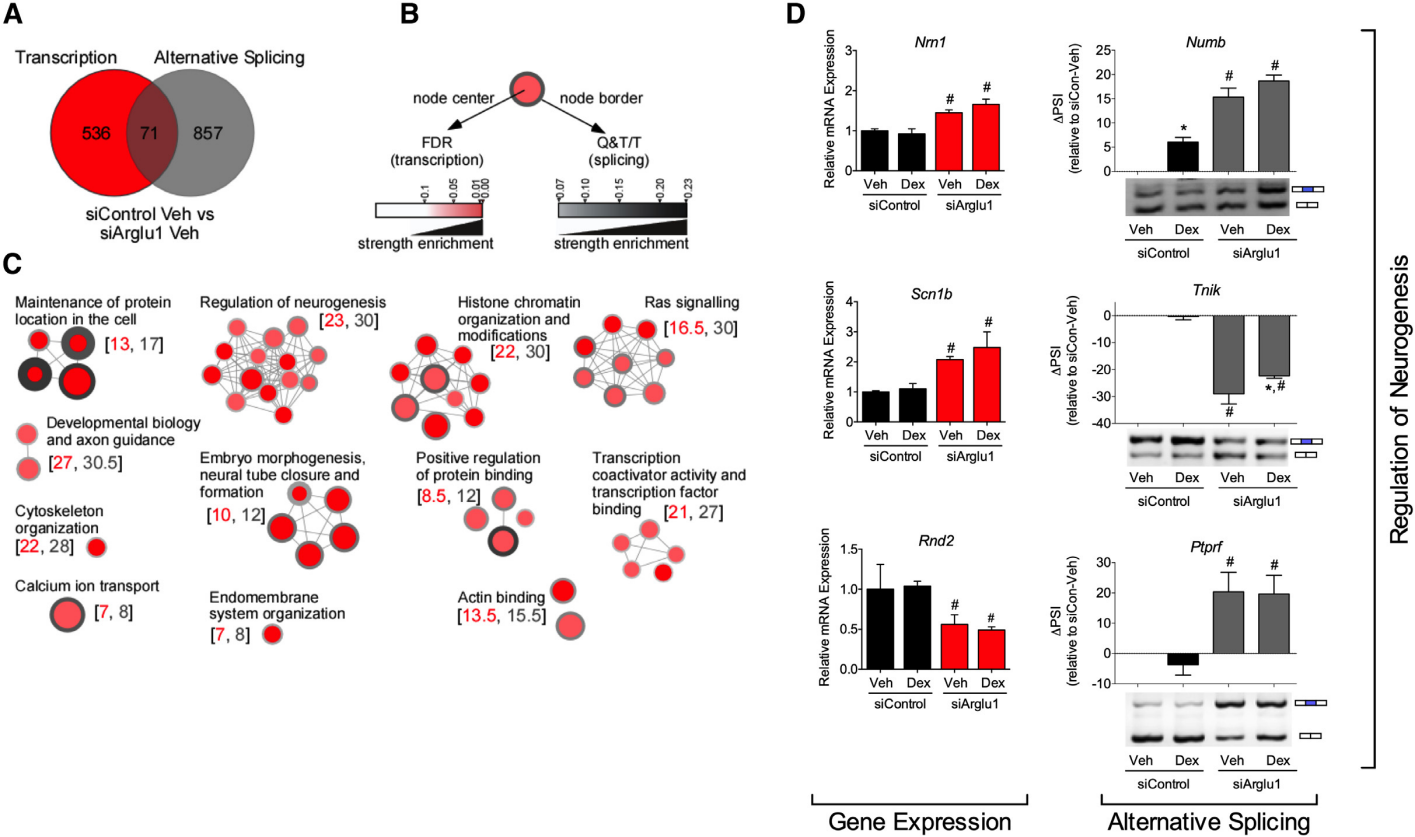
ARGLU1 basally regulates biological processes through modulation of transcription and AS of separate genes. (**A**) Venn diagram showing limited overlap in genes alternatively spliced (PSI ≥ 15) and transcriptionally regulated (siArglu1/siControl log_2_ ratio ≤−0.81 or ≥0.81) by ARGLU1 in N2a cells in the absence of a ligand. Genes having more than one splicing event were only counted once. Legend in (**B**) for pathways demonstrating overlap between those basally regulated by ARGLU1 in alternative splicing vs. transcription (siArglu1 versus siControl). (**C**) Node size is proportional to the number of differentially expressed genes in a pathway (corrected by the size of the pathway). All these pathways have a significant enrichment FDR equal or less than 0.05 for both the alternative splicing and transcription gene lists. Related pathways are grouped together under a common label to form modules. The numbers in brackets correspond to the median number of genes for transcription (left) and alternative splicing (right) contained in each pathway module. FDR, false discovery rate. Q&T/T, number of genes in the overlap for each pathway corrected by the size of the pathway. (**D**) Genes involved in regulation of neurogenesis were altered upon ARGLU1 knockdown in N2a cells at both transcriptional and AS levels. One-step RT-PCR validation of splicing events with a ΔPSI ≥15 is shown. A representative image is shown below the Image J quantification of the blot. Data represent the mean ± SEM (*n* = 3). *P < 0.05 versus respective Veh, ^#^*P* < 0.05 versus respective siControl; ANOVA followed by Neuman–Keuls test. See also Supplementary Figure S9.

Analysis of RNA-seq data for alternative splicing changes showed ARGLU1 controls basal AS patterns in N2a cells. Alternative splicing was assessed using the established *vast-tools* bioinformatics pipeline (22) from which the ‘percent spliced in’ (PSI) metric was generated. For our analysis, only splicing events that showed changes in PSI (delta PSI, ΔPSI) of ≥15 were analyzed further. Knockdown of ARGLU1 in vehicle treated cells resulted in 1129 differential AS events (within 928 genes) compared to siControl (|ΔPSI| ≥ 15 calculated as siArglu1 Veh PSI—siControl Veh PSI) (Supplementary Table S3), indicating that ARGLU1 regulates AS patterns in N2a cells. The majority of AS events affected by ARGLU1 belonged to the simple and complex cassette exon category (Figure 3D). When comparing the direction of the change in cassette exons within the differential AS events, loss of ARGLU1 promoted exon inclusion or skipping in 56% and 44% of events, respectively (Figure 3D, Supplementary Table S3). One-step RT-PCR was used to validate 20 AS events showing differential inclusion levels upon ARGLU1 knockdown (Figures 4D, Supplementary Figure S9B). The ΔPSI values measured from RNA-seq and one-step RT-PCR were highly correlated (*r* = 0.916, Supplementary Figure S9C). Similar results were observed with two independent siRNAs targeting ARGLU1, suggesting the changes we observed were due to on-target effects (Supplementary Figure S9D). These data support a basal role for ARGLU1 in the regulation of alternative splicing by binding both to RNA and to other key components of the spliceosome complex.

Next, we examined Dex-responsive AS events. There were 426 AS events that showed absolute ΔPSI of ≥15 in response to Dex in the siControl group (ΔPSI calculated as siControl Dex PSI – siControl Veh PSI) (Supplementary Table S3). Selected AS events modulated by Dex were validated in three independent experiments (*i.e., Klf15, Mpdz, Brwd3, Tmtc1*, Supplementary Figure S9B). Remarkably, only 28 of these Dex-induced events were preserved in the absence of ARGLU1 (*i.e*., still present in ΔPSI = siArglu1 Dex PSI – siArglu1 Veh PSI) suggesting that ARGLU1 was required for this Dex-mediated action. Without ARGLU1, Dex-treatment promoted the alternative splicing of a different set of events (Supplementary Table S3). To assess whether GR is itself associated with spliceosomal proteins we turned to our BirA*-GR IP-MS data. Of the 1074 proteins that were enriched in BirA*-GR+Dex, 240 proteins were also enriched in the BirA*-ARGLU1+Dex. Gene ontology analysis of these 240 common proteins found that proteins in transcription factor and spliceosomal complexes were over-represented (Supplementary Figure S8, overlap). Importantly, ARGLU1 was found to be a substrate of BirA*-GR, suggesting that these two proteins are in close proximity (within 10 nm) inside the cell (Supplementary Table S5). Further studies are required to determine whether the Dex-mediated splicing is functionally (directly or indirectly) linked to ARGLU1. Nonetheless, our data strongly support a role for ARGLU1 in maintaining proper basal AS in N2a cells.

To assess whether ARGLU1 associates with RNA in a cellular context, we conducted formaldehyde cross-linking/RNA-immunoprecipitation experiments using nuclear lysates from N2a cells expressing FLAG-tagged ARGLU1 (FL), ARGLU1^N-term^, or ARGLU1^C-term^ and quantified bound pre-mRNA using RT-qPCR. Our results showed that full-length ARGLU1 and ARGLU1^N-term^ (but not ARGLU1^C-term^) pulldowns enrich *Lpin1* and *Bin1* pre-mRNA (both are alternatively spliced in an ARGLU1-dependent manner and contain G-rich sequences near ARGLU1-dependent splice sites) (Supplementary Figure S9E). In contrast, *Brwd3* pre-mRNA (undergoes ARGLU1-dependent AS but does not contain G-rich sequences near splice sites) is not enriched (Supplementary Figure S9E). Together the RNAcompete, splicing, and RNA pulldown data suggest that ARGLU1 may directly regulate alternatively splicing of a subset of pre-mRNA. Computational analysis did not detect global enrichment of ARGLU1 RNA binding sites in regions (±300 bp) surrounding alternatively spliced exons. Thus, it is likely that ARGLU1 mediates alternative splicing by both direct and indirect mechanisms.

### ARGLU1 regulates gene expression and AS on distinct genes within similar networks

It is widely accepted that splicing and transcription are temporally coupled (40). To explore whether ARGLU1 facilitates the coupling of these two processes, we compared the genes affected by ARGLU1 in each category. Interestingly, out of the 607 transcriptionally regulated and 928 alternatively spliced genes that were affected by ARGLU1 knockdown (siArglu1 versus siControl), only 71 genes were overlapping (Figure 4A, Supplementary Table S4). This limited overlap strongly suggests that ARGLU1 has two distinct roles within cells. On one set of genes, it acts solely as a transcriptional co-activator and for a separate set of genes, it behaves as a splicing modulator.

Strikingly, although differentially expressed and alternatively spliced genes were largely unique, they were frequently enriched within the same pathways including ‘regulation of neurogenesis’ and ‘histone chromatin organization and modifications’ (Figure 4B, C). These data suggest that although ARGLU1 basally regulates the AS and expression of distinct genes, these genes share a similar functional classification. For example, knockdown of ARGLU1 significantly altered the mRNA expression of genes such as *Nrn1, Scn1b*, and *Rnd2* involved in neuronal differentiation and cell morphogenesis and increased exon skipping of *Numb, Tnik* and *Ptprf*, alternatively spliced genes involved in neuronal morphogenesis (Figure 4D).

### Loss of ARGLU1 is embryonic lethal in mice and causes neurodevelopmental defects in zebrafish

**Figure 5.**
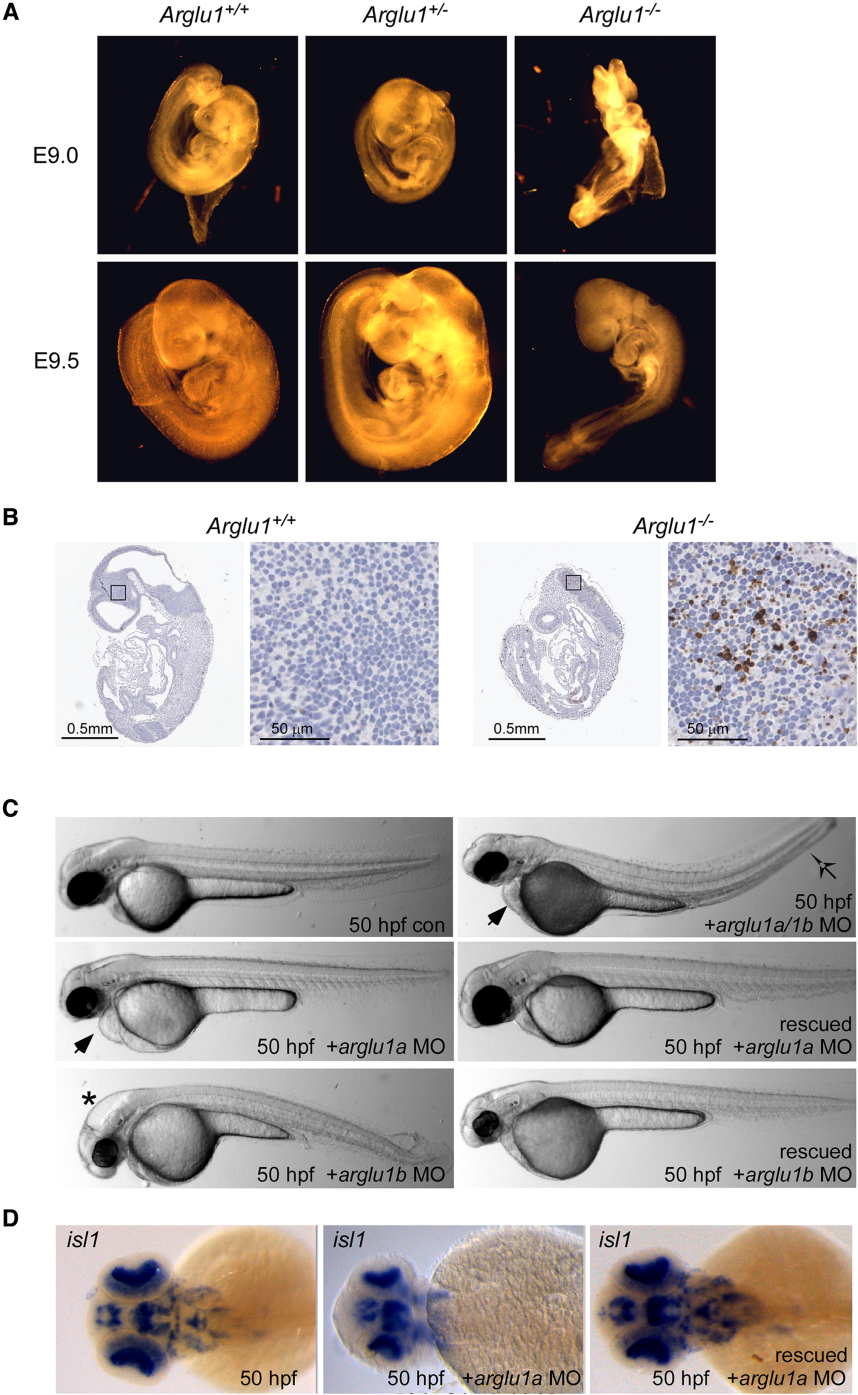
ARGLU1 regulates vertebrate nervous system development. (**A**) Mouse embryos from the *Arglu1^+/−^* x *Arglu1^+/−^* parent crosses were dissected and visually examined at E9.0 and E9.5. *Arglu1^+/-^* embryos appeared phenotypically normal whereas *Arglu1^−/−^* embryos had an overall developmental delay by approximately 0.5 days. (**B**) Cleaved caspase-3 was measured by immunohistochemistry in *Arglu1^+/+^* and *Arglu1^−/−^* embryos from E9.0 and E9.5, respectively. These time points were chosen to allow cleaved caspase-3 comparisons under matched developmental stages for each genotype. (**C**) Zebrafish embryos were injected with the indicated morpholino antisense oligonucleotide (MO) alone or in the presence of the indicated rescue mRNA constructs and visualized at 50 hpf. Symbols represent heart edema (
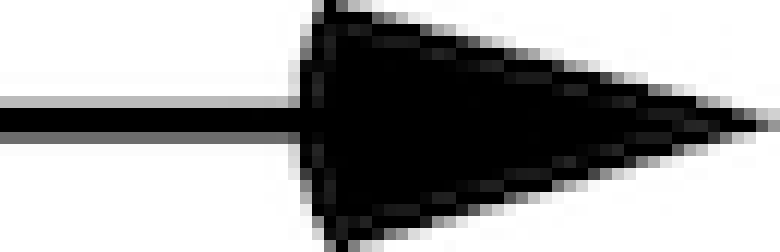
), expanded brain ventricle (*) and curved body axis (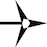). (**D**) RNA *in situ* hybridization assays monitoring islet-1 expression in 50 hpf embryos. p53-MO-injected control, arglu1a morphant embryo, and arglu1a morphant embryo rescued through co-injection of *arglu1a* mRNA are shown. Hpf, hours post fertilization. See also Supplementary Figures S10 and S11.

To investigate the physiologic role of ARGLU1 *in vivo*, we attempted to generate a whole-body ARGLU1 knockout mouse. Heterozygous Arglu1 mice (*Arglu1^+/−^* also known as *Arglu1^tm1a/+^*) were generated by morula aggregation using commercially available ES cell lines (EUCOMM, details provided in the Extended methods and Supplementary Figure S10). Male and female heterozygous *Arglu1^+/–^* mice appeared phenotypically normal. Breeding of heterozygous *Arglu1^+/−^* mice did not produce any homozygous knockout mice (out of 200 pups) suggesting that *Arglu1^−/−^* mice are embryonic lethal. To begin to identify the developmental defects, we performed timed pregnancy experiments. Genotyping of the yolk sacs detected *Arglu1^−/−^* embryos at E9.0 and E9.5 but not at E12.5 (data not shown). Gross morphologic examination of these mutant embryos revealed an overall developmental delay of ∼0.5 days at E9.0 and E9.5 (Figure 5A). This mid-gestation lethality is consistent with a generalized growth defect and demonstrates that ARGLU1 is indispensable for embryonic development before E9.5. Moreover, staining for the apoptotic marker, cleaved caspase-3 (Figure 5B), demonstrated increased apoptosis in the developing brain of *Arglu1^−/−^* embryos when compared to *Arglu1^+/+^* (Figure 5B). These data suggest that ARGLU1 is required to maintain cellular integrity during CNS development.

The high evolutionary conservation of ARGLU1 allowed us to utilize the zebrafish *Danio rerio* as a second model organism to study ARGLU1’s role *in vivo*. Interestingly, unlike mammalian ARGLU1, which is encoded by only one gene, zebrafish possess two ARGLU1 paralog genes encoded on separate chromosomes. The sequence identities of ARGLU1a and ARGLU1b to human ARGLU1 are 84% and 87%, respectively. The expression of *arglu1a* and *arglu1b* was examined by whole-mount RNA *in situ* hybridization at 24 and 48 hours post-fertilization (hpf). Diffuse expression of both isoforms was observed throughout the brain at each time point (Supplementary Figure S11A–D). These results are consistent with the high CNS expression of *Arglu1* in mice.

To examine the effect of loss of ARGLU1 proteins in zebrafish, we injected single cell-staged embryos with translation blocking morpholinos (MO) targeting *arglu1a* and *arglu1b*, alone or in combination. To minimize the off-target effects of morpholino injections mediated by the p53 apoptotic pathway, p53 MO was co-injected (41). At 50 hpf, p53 MO injected control embryos appeared phenotypically normal (Figure 5C); whereas, more than 70% of arglu1a MO-injected embryos exhibited heart edema and decreased brain size (*n* = 85, Figure 5C). Interestingly, arglu1b MO-injected animals (more than 90%) showed a different morphant phenotype, with expanded brain ventricles and a curved body axis (*n* = 85, Figure 5C). Co-injection of arglu1a and 1b MO together led to a more severe phenotype, where animals exhibited abnormal brain development, heart defects and a curved body axis (*n* = 30, Figure 5C). All embryos which had the morphant phenotype showed impaired movement without lethality up until the feeding stage (8–9 days) where they were unable to feed and died. To confirm that the observed results were not due to off-target effects of MO injection, we performed rescue experiments by co-injecting the arglu1a and arglu1b MOs with *in vitro* transcribed *arglu1a* or *arglu1b* mRNA, respectively (Figure 5C). *arglu1a* and *arglu1b* mRNA was able to rescue the morphant phenotypes in 65% of the embryos (*n* = 60, Figure 5C).

To better delineate the brain defect in arglu1a MO embryos, we performed RNA *in situ* hybridization for *islet-1*, a LIM homeodomain-containing transcription factor which acts as an early marker of neuronal differentiation. At 50 hpf, p53 MO injected control animals showed the characteristic *islet-1* expression pattern in the brain (Figure 5D), whereas arglu1a MO injected embryos had strikingly reduced *islet-1* expression, indicating possible delay or disruption of neuronal differentiation (Figure 5D). Validating the on-target activity of the MO, co-injection of *arglu1a* mRNA rescued expression of *islet-1* in these embryos (Figure 5D). From these studies, our data suggest that ARGLU1 proteins in zebrafish play an important role in normal development.

## DISCUSSION

### ARGLU1 is an evolutionarily conserved NR coactivator and AS modulator

We detail the discovery and characterization of ARGLU1 as a highly conserved dual function protein working as both a nuclear receptor coactivator and AS effector. ARGLU1 represents a new member within the diverse family of NR coregulatory proteins, since it shares no sequence similarity among other coregulators identified to date and contains no consensus mammalian RNA binding domains. Consistent with our findings, a functional genomics screen performed in mouse ES and N2a cells recently confirmed that knockdown of ARGLU1 affects AS (42). Our data show that deletion of ARGLU1 was embryonic lethal in mice between E9.5 and E12.5 coinciding with increased apoptosis in mouse neurons, and in severe developmental defects in the CNS and heart of zebrafish. Biochemical studies determined that ARGLU1 interacts with GR through its C-terminal domain; whereas, the N-terminal domain mediates interactions with splicing factors and contributes to both basal and GC-dependent splicing outcomes. Remarkably, only 7.5% of the genes differentially alternatively spliced by ARGLU1, were also transcriptionally regulated by ARGLU1. These findings strongly implicate ARGLU1 as a dual-function protein, utilizing its two distinct structural domains to regulate gene expression at two levels (transcription and pre-mRNA splicing). Pathways affected by loss of ARGLU1 included regulation of neurogenesis and histone chromatin remodeling, which may have contributed to the developmental defects observed in the mouse and zebrafish loss of function models.

Consistent with many NR coregulators, ARGLU1 coactivates multiple receptors in both a ligand dependent and independent fashion. In agreement with our discovery of ARGLU1 as a GR coactivator, Zhang and colleagues showed that ARGLU1 interacts with a mediator complex subunit and is required for amplifying ERα–mediated gene transcription. ARGLU1 was also recently shown to participate in the regulation of its own splicing via the presence of an ultraconserved element (43). Among the receptors that showed ligand dependence for ARGLU1, GR was the most highly dependent followed by ERα and several others to a much lower extent. The synergy in the transcriptional output ARGLU1 with other GR coactivators (TIF2 and PGC1α) is consistent with its function as a coactivator. Such effects have been observed previously between coactivators and NRs (29,44). From our BioID data we found that GR and ARGLU1 are in close proximity as BirA*-GR biotinylated ARGLU1. In contrast, we did not observe biotinylation of GR with BirA*-ARGLU1. The lack of complementarity in the results may be due to the relative topology of the BirA* tag on each of the proteins in three-dimensional space. Nonetheless, in the presence of Dex, there were 240 proteins that were shared between GR and ARGLU1. We mapped these proteins to the curated nuclear receptor co-regulator list on the NURSA website (www.nursa.org) and discovered 22 proteins in common including MED1 and TIF2/NCOA2. Thus, in addition to GR and ARGLU1 being in close proximity in the cell, these data suggest they may be in a protein complex with other known nuclear receptor coregulators that help mediate a full Dex response. A recent analysis of the GR protein interactome from mouse liver identified ARGLU1 as a protein enriched following GR pull-down compared to IgG control, further supporting the idea that the two proteins are present in a complex (45).

The two domains of ARGLU1 have opposing charges with positively charged arginine residues at the N-terminus and an abundance of negatively charged glutamate residues at the C-terminus. Proteomic analysis indicated that the N-terminal end was responsible for interacting with splicing factors. Our findings are also supported by a proteomics study of the human spliceosome that identified ARGLU1 (FLJ10154) as one of the proteins repeatedly co-purified with the U2 related spliceosomal complex (46). Intriguingly, although ARGLU1 contains no consensus mammalian RNA binding motifs, the highly enriched segment of arginines at the N-terminus is consistent with the mechanism by which some viral proteins bind RNA (i.e., HIV Rev and Tat proteins) (47). ARGLU1 was also recently shown to interact with the splicing factor SRSF2 in a co-IP assay and these studies suggest that ARGLU1 may influence AS by competing with SRSF2 for RNA binding (42).

### Stress hormone dependent AS on a genome-wide scale

NR co-regulator studies have traditionally examined the interplay of NRs and their coactivators in the initiation of gene transcription and their interaction with chromatin; with much less attention paid to the post-transcriptional role of NR coregulators in signaling (48). Splicing is orchestrated by a multi-protein complex composed of five small ribonucleoprotein particles and up to ∼300 splicing factors (49,50). It is estimated that ∼90% of human pre-mRNAs are alternatively spliced (51,52). The regulation of AS is known to occur co-transcriptionally and/or post-transcriptionally depending on the gene (53–55).

In the context of NR signaling, steroid NRs and their coactivators have been implicated in AS with the idea that activated steroid hormone receptors control gene transcription and affect splicing decisions in a promoter-dependent manner by recruiting a set of transcriptional coregulators that participate in the splicing decisions of the newly formed transcripts (13–15). For example, PPARγ coactivator, PGC1α, was shown to influence the AS of the fibronectin minigene when PPAR/RXR binding sites were present in the promoter (16). Similarly, coregulators of steroid receptors, CAPER and CoAA, were found to play a role in the pre-mRNA processing of various minigenes in response to ligand (14,56,57). In cells, CAPERα was found to coactivate the progesterone receptor and alter the splicing of endogenous *Vegf* (56). In these models, the influence of the coregulatory protein on splicing was intimately tied to transcriptional activation, generally in response to hormone through the incorporation of the MMTV promoter upstream of the minigene construct. These results invoke the kinetic model of co-transcriptional splicing in which activation of transcription by the NR is a co-requisite to its role in splicing.

In contrast, our data suggest that the above model is not applicable in a general context. When examined on a genome-wide scale, we find that among the genes that were significantly altered in response to Dex, there was no overlap between genes transcriptionally regulated and those that were alternatively spliced (though we acknowledge that only seven genes met the criteria for statistical significance for transcription in siControl Dex vs. Veh). This finding supports the idea that changes in splicing, in general, do not affect overall steady-state transcript levels from the same gene. However, we cannot rule out the possibility that in some cases, changes in the stability of the alternative transcript could be playing a role. Other groups have previously demonstrated a role for GC regulation of AS on individual genes (58–60); however, to our knowledge, our study is the first to examine the global effect of GC signaling on AS. Although these findings were unexpected based on historical minigene data, our observations are in agreement with a study in which an exon array was used to probe the role of ERα and ERβ in estradiol-induced AS (61). This study found that ∼67% of genes that were alternatively spliced after a 3 h treatment were not transcriptionally altered (61). More recently, RNA-seq analysis of breast cancer cells treated with a synthetic progestin found 254 genes alternatively spliced after a 6 h treatment, 74% of which were not transcriptionally regulated (62).

The degree to which ARGLU1 is interfacing specifically with GR to regulate genes is, as yet, not clear and will require further study. In the liver, it was recently shown that ARGLU1 is pulled down as part of the GR protein interactome (45). Our BirA* and ChIP data support the close proximity of GR and ARGLU1 within the cell, but fall short of proving the two are acting within the same complex. Therefore, it is possible that the overlapping requirement for ARGLU1 for several Dex-mediated transcriptional and splicing events is co-incidental based on their shared roles in common pathways. However, we do note that of the genes significantly induced by Dex in N2a cells (siControl), the magnitude of the fold-induction was diminished for all seven genes by the absence of ARGLU1 (Supplementary Table S1). Likewise, of the 426 splicing events that were alternatively spliced in response to Dex, only 28 remained Dex-responsive in the absence of ARGLU1. Together, these data suggest that in N2a cells, the presence of ARGLU1 influences GC signaling outcomes.

## SUMMARY

Overall, these data support two distinct functional roles for ARGLU1 in development, one involved in transcription and the other in AS. It is not yet clear whether the developmental defects observed in mice and zebrafish with loss of ARGLU1 are due to the loss of its transcriptional or splicing role, but we anticipate that both will be important based on our bioinformatics analysis in neural cells that found that developmental pathways were enriched in both the transcriptionally regulated and alternatively spliced gene lists (Figure 4). Our data also suggest that stress hormone-induced AS is a layer of gene regulation that deserves further study, though we acknowledge that we do not yet know whether ARGLU1 plays a direct or indirect role in this context. Because the brain is prone to large changes in alternative splicing, this result may be particularly important with respect to stress-induced cognitive dysfunction and provide a new angle from which to explore the molecular mechanisms of hormone function in disease states.

## DATA AVAILABILITY

Raw RNA-seq data have been deposited with GEO omnibus under accession number GSE85197. The mass spectrometry proteomics data have been deposited to the ProteomeXchange Consortium (http://proteomecentral.proteomexchange.org) via the PRIDE partner repository (63) with the dataset identifier PXD012406 and PXD012555.

## Supplementary Material

Supplementary Data
