# Supplementary material for: ARGLU1 is a transcriptional coactivator and splicing regulator important for stress hormone signaling and development
Source: Nucleic Acids Res. 2019 Jan 30;47(6):2856–70. doi: 10.1093/nar/gkz010 (PMC6451108; doi:10.1093/nar/gkz010)
Supplement: Supplementary Data [file gkz010_supplemental_files.zip › ARGLU1_Magomedova_SupData_Dec20_Jan13LM.docx]

**SUPPLEMENTAL TABLES (uploaded as excel files)**

Table S1. N2a RNA-seq gene expression data set.

N2a RNA-seq data for genes which showed significant differential gene expression following ARGLU1 knockdown (p<0.05 by edgeR analysis). Raw and analyzed data are presented in separate tabs. Some columns are hidden to increase readability.

Table S2. Gene ontology functional classes of ARGLU1 regulated genes in N2a cells.

g:Profiler pathway enrichment analysis of differentially expressed genes (p<0.05 by edgeR) of untreated N2a cells lacking ARGLU1.

Table S3. N2a RNA-seq alternative splicing data set.

Only events with ∆PSI of ≥ 15 for the indicated condition are shown. Event complexity refers to the percentage of reads that do not come from the C1A, AC2, C1C2 exon junctions. S is a simple event with ≤5% of total reads being complex, while C1, C2, and C3 have an increased proportion of complex reads. Other codes refer to additional types of AS events, where MIC refers to microexons (≤ 15 nucleotides). Some columns are hidden to increase readability.

Table S4. N2a genes showing transcription and splicing overlap following ARGLU1 knockdown.

N2a RNA-seq data for genes which showed significant (p<0.05 by edgeR analysis) changes in basal expression level upon ARGLU1 knockdown and overlapping genes which showed splicing changes of ∆PSI of ≥ 15 (calculated as siArglu1 PSI – siControl PSI). Event complexity refers to the percentage of reads that do not come from the C1A, AC2, C1C2 exon junctions. S is a simple event with ≤5% of total reads being complex, while C1, C2, and C3 have an increased proportion of complex reads. Other codes refer to additional types of AS events, where MIC refers to microexons (≤ 15 nucleotides). A total of 71 overlapping genes were identified. Some columns are hidden to increase readability.

Table S5. Proteins identified by mass spectrometry using BioID in HEK293 cells.

Lists of proteins identified by BioID using BirA*-ARGLU1 or BirA*-GR as bait. Cells transfected with BirA* alone were used as negative controls. Cells were treated with 100 nM dexamethasone (Dex) for 24 h. The normalized MS intensities (iBAQ) obtained with the indicated BirA*-tagged bait or the BirA* alone are shown for each of the identified proteins. Raw and analyzed data are presented in separate tabs. Some columns are hidden to increase readability.

Table S6. Proteins identified by mass spectrometry using RIME in N2a cells.

Lists of proteins identified by RIME (rapid immunoprecipitation of endogenous protein complexes) using anti-IgG, anti-ARGLU1 and anti-GR antibodies following 1 h of 100 nM dexamethasone (Dex) treatment. The normalized MS intensities (iBAQ) obtained with the indicated antibody are shown for each of the identified proteins. Raw and analyzed data are presented in separate tabs.

**SUPPLEMENTAL FIGURES**





**Figure S1:** **A screen for new modulators of GR activity identified ARGLU1, a nuclear protein from a human brain cDNA library, as a GR coactivator, related to Figure 1.** (**A-B**) cDNA pools from a human brain library were co-transfected into HEK293 cells with GAL4-hGR, UAS-luciferase and β-galactosidase. Cells were treated with 300 nM cortisol (Cort) and the luciferase signal was compared to no library control (empty vector). The coactivator (CoA) TIF2 and corepressor (CoR) RIP140 were used as positive controls. The positive hit shown in light purple (>2-fold change) was first screened at 50 clones per well (**A**), then 12 clones per well (not shown), and finally 1 clone per well (**B**). The clone in light purple was identified as ARGLU1. (**C**) HEK293 cells transfected with the GAL4-GR/UAS-luc reporter with increasing amounts of CMX-ARGLU1 and 300 nM cortisol (Cort). (**D**) HEK293 cells transfected with the CMX-hGR/MMTV-luc reporter with a fixed amount of CMX-ARGLU1 (15 ng/well) and increasing concentrations of cortisol. Data represent the mean ± SD, ANOVA followed by Newman-Keuls, *p<0.05 Cort vs respective Veh; #p<0.05 vs 0 ng.


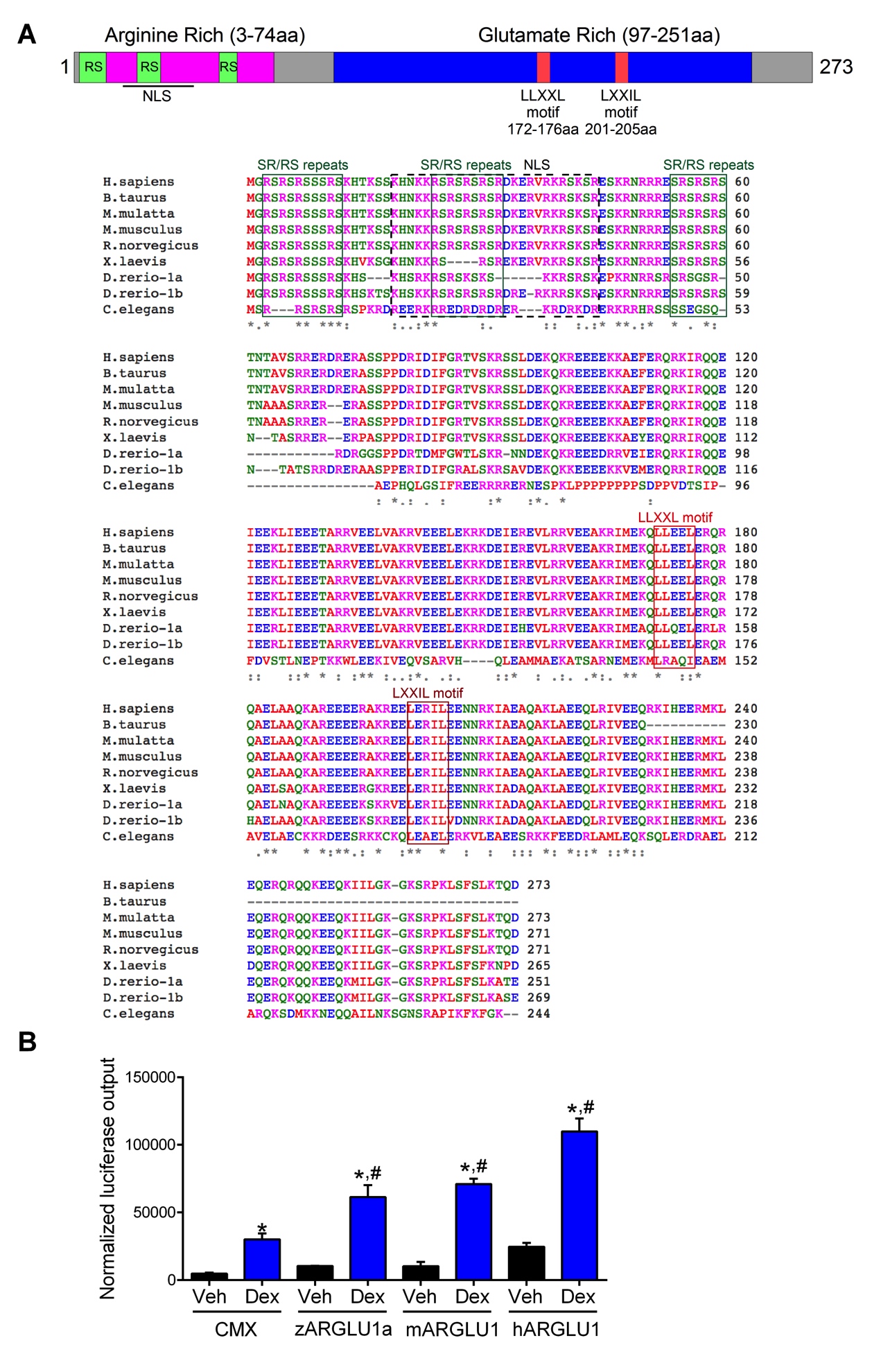


**Figure S2: ARGLU1 is a highly evolutionary conserved GR coactivator, related to Figure 1.**

(**A**) Amino acid sequence alignment between species. ARGLU1 has two distinct regions: the N-terminus which is rich in positively charged arginine amino acids, and the C-terminus which is composed of glutamate rich amino acids. The arginine-rich region is also enriched in the SR/RS-repeats (boxed). Bipartite nuclear localization sequence (NLS) is depicted by a dashed box. Two putative NR interaction domains, LLXXL and LXXIL motifs, were identified by visual examination: L, leucine; I, isoleucine; X, any amino acid (boxed). Sequence alignment was performed using ClustalW. (**B**) ARGLU1’s GR coactivation function is preserved across species. HEK293 cells were co-transfected with GAL4-GR and UAS-luciferase together with 15 ng of zebrafish (zARGLU1a), mouse (mARGLU1) or human (hARGLU1) ARGLU1. Six hours post-transfection, cells were treated with 100 nM dexamethasone (Dex) and harvested for luciferase assay 16 h later. β-galactosidase was used to normalize for transfection efficiency. When compared to the CMX group all three ARGLU1 orthologues were able to significantly induce GAL4-GR activity in response to Dex. Normalized luciferase output = luciferase light units/β-galactosidase*time. Data represent the mean ± SD. *p<0.05 Dex group vs respective Veh; #p<0.05 vs CMX-Dex. ANOVA followed by Newman-Keuls test.


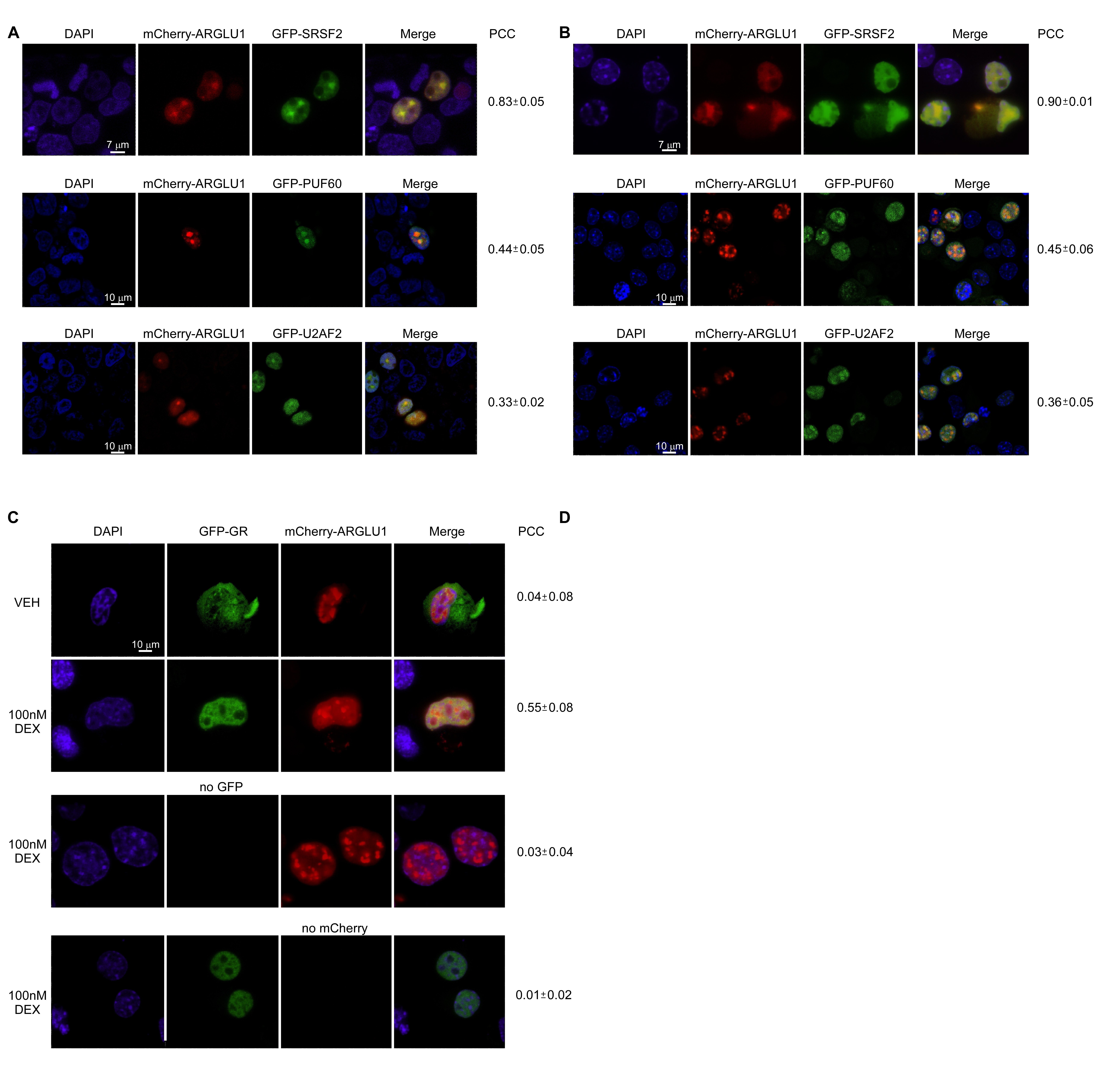


| **HEK293 cells** | | | | | |
| --- | --- | --- | --- | --- | --- |
| **mCherry** | **GFP** | **Dex** | **Cell #** | **Pearson**  **Correlation**  **Coefficient**  **Mean** | **PCC SEM** |
| ARGLU1 | hGR | - | 52 | -0.03 | 0.05 |
|  | hGR | + | 31 | 0.52 | 0.07 |
|  | SRSF2 | - | 15 | 0.83 | 0.05 |
|  | PUF60 | - | 12 | 0.44 | 0.05 |
|  | U2AF2 | - | 9 | 0.33 | 0.02 |
| SRSF2 | hARGLU1 | - | 6 | 0.81 | 0.01 |
| ARGLU1 | - | + | 6 | 0.10 | 0.05 |
| - | GFP | + | 20 | 0.01 | 0.02 |
| **N2a cells** | | | | | |
| **mCherry** | **GFP** | **Dex** | **Cell #** | **Pearson Correlation Coefficient Mean** | **PCC SEM** |
| ARGLU1 | hGR | - | 3 | 0.04 | 0.08 |
|  | hGR | + | 3 | 0.55 | 0.08 |
|  | SRSF2 | - | 8 | 0.90 | 0.01 |
|  | PUF60 | - | 33 | 0.45 | 0.06 |
|  | U2AF2 | - | 19 | 0.36 | 0.05 |
| SRSF2 | hARGLU1 | - | 8 | 0.88 | 0.02 |
| ARGLU1 | - | + | 16 | 0.03 | 0.04 |
| - | GFP | + | 21 | 0.01 | 0.02 |

**Figure S3: Immunofluorescence co-localization studies of ARGLU1 with splicing factors, related to Figure 1.**

Co-localization studies of mCherry-ARGLU1 (red) and GFP-SRSF2, GFP-PUF60, GFP-U2AF2 or GFP-GR (green) in (**A**) HEK293 and (**B, C**) Neuro-2a cells. DAPI (blue) was used to stain the nuclei. Pearson’s correlation coefficient (PCC) for localization overlap is listed on the right of the corresponding images (mean ± SEM) and summarized in (**D**).


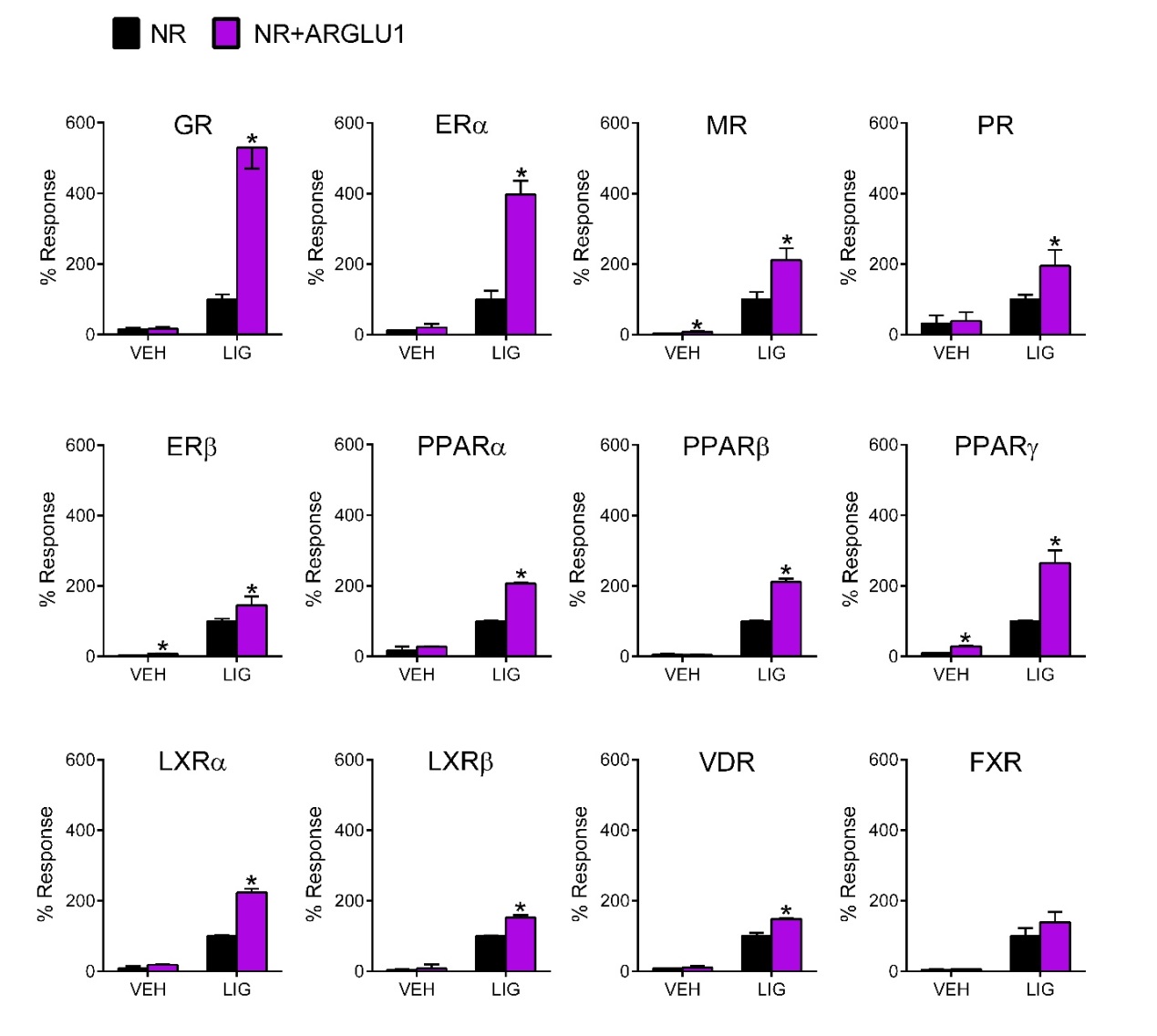


**Figure S4:** **ARGLU1 activation of nuclear receptors, related to Figure 2.**

Various GAL4-nuclear receptor (NR) fusion proteins were co-transfected with the UAS-luciferase system and 15 ng ARGLU1 into HEK293 cells. Six hours later, cells were treated with 300 nM cortisol (hGRα), 10 nM aldosterone (hMR), 10 nM progesterone (hPR), 1 nM 17β-estradiol (hERα and hERβ), 500 nM T0901317 (hLXRα/hLXRβ), 500 nM WY14643 for (hPPARα), 25 nM GW1516 (hPPARδ), 50 nM rosiglitazone (hPPARγ), 10 μM CDCA (hFXR) and 0.5 nM 1,25(OH)_2_ vitamin D_3_ (hVDR). All ligands were dosed at the receptor’s EC_50_ in this system. Data represent the mean ± SD (n=3). *p<0.05 NR+ARGLU1 vs NR; Student’s t-test.

**
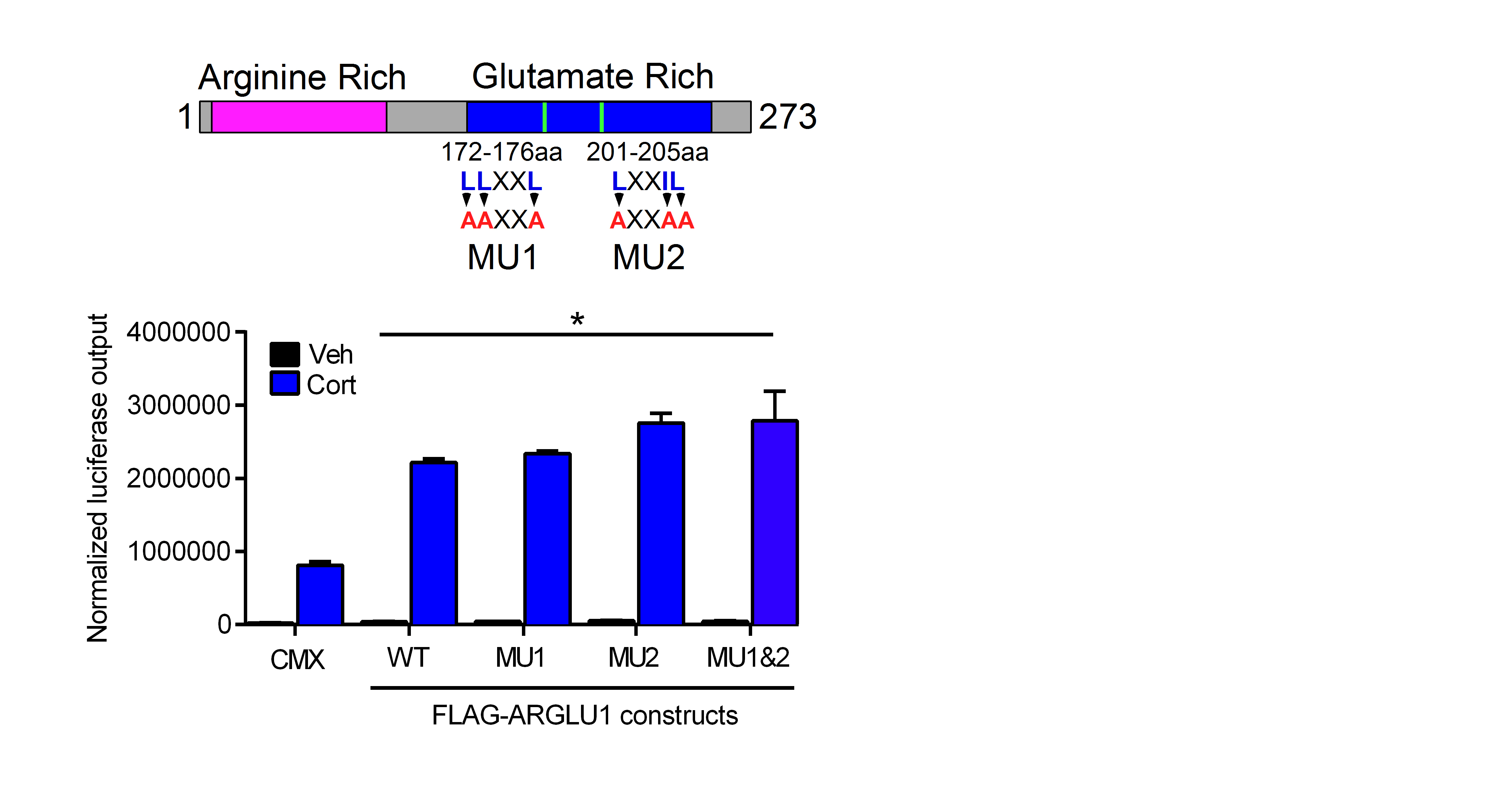
**

**Figure S5: ARGLU1 activation of GR is not dependent on the individual LXXLL motifs, related to Figure 2.**

Top - Schematic diagram of ARGLU1 mutants. Bottom - GAL4-GR was cotransfected into HEK293 cells with the UAS-luciferase system and 15 ng of the indicated ARGLU1 LLXXL mutants, followed by administration of EtOH (Veh) or 300 nM cortisol. CMX was used as a control. Normalized luciferase output = luciferase light units/β-galactosidase*time. Data represent the mean ± SD (n=3). *p<0.05 vs CMX-Cort; ANOVA followed by Newman-Keuls test.





**Figure S6: Loss of ARGLU1 significantly alters basal gene expression program in Neuro2a cells, related to Figure 2.**

Neuro-2a cells were transfected with 30 pmol of siControl and siArglu1using RNAiMax for 48 h and then treated with vehicle (EtOH) or 100 nM Dex for 4 h before RNA or protein extraction. (**A**) Quantitative PCR of *Arglu1* and *Gr* mRNA normalized to cyclophilin. (**B**) ARGLU1 protein expression in whole cell lysates analyzed by Western blot (n=3). (**C**-**D**) MA plot showing the distribution of read counts (x-axis) against p value score (defined in methods from edgeR analysis) for each gene. Orange - top 100 genes; Red – genes validated by qPCR. (**E**) Volcano plot of log_2_ fold change (log_2_FC) versus p value score (defined in methods from edgeR analysis) for differentially expressed genes. Orange - top 100 genes. Red – genes validated by qPCR. (**F**) Correlation between fold change values from qPCR and RNA-seq data are shown without *Pnmt*. Inclusion of *Pnmt* yielded a correlation coefficient of r = 0.9515. (**G**) qPCR validation of *Per1* gene expression. (**H**) ChIP of GR and ARGLU1 at the GRE of the *Per1* gene. (**I**) ChIP of IgG at each of the loci tested. (**J**) qPCR validation of RNA-seq data was performed on non-pooled samples. Data represent the mean ± SEM (n=3). *p<0.05 vs respective Veh, #p<0.05 vs siControl; ANOVA followed by Newman-Keuls test.

**
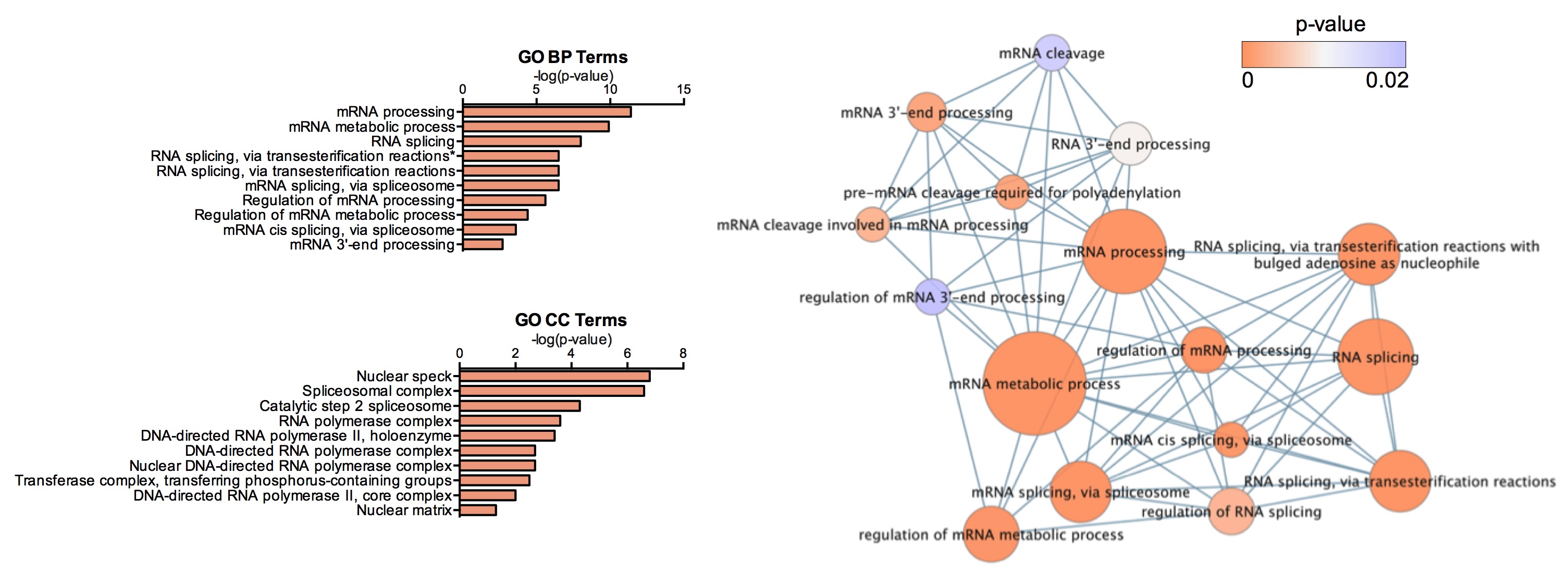
**

**Figure S7: ARGLU1 interacts with splicing factors in N2a cells, related to Figure 3.**

Immunoprecipitation of ARGLU1 from N2a cells treated with 100 nM Dex using rapid immunoprecipitation mass spectrometry of endogenous proteins (RIME) for analysis of chromatin complexes. RIME identified 1538 unique proteins. Of those proteins, 150 were detected in the ARGLU1 + Dex sample and not detected in the IgG controls. The list was submitted to g:Profiler. to test for Gene Ontology enrichment (BP – Biological process and CC – Cellular Compartment). The conditions were set such that the minimum overlap is 2 proteins, and the size of the functional category is between 3 to 500 proteins. The statistical threshold was calculated using the Benjamini-Hochberg FDR method. The top 10 most significant GO BP and GO CC terms were represented using the negative logarithm of their p-value. The GO BP Enrichment Map was drawn in Cytoscape’s Enrichment Map plug-in using the g:Profiler GO BP output.

**
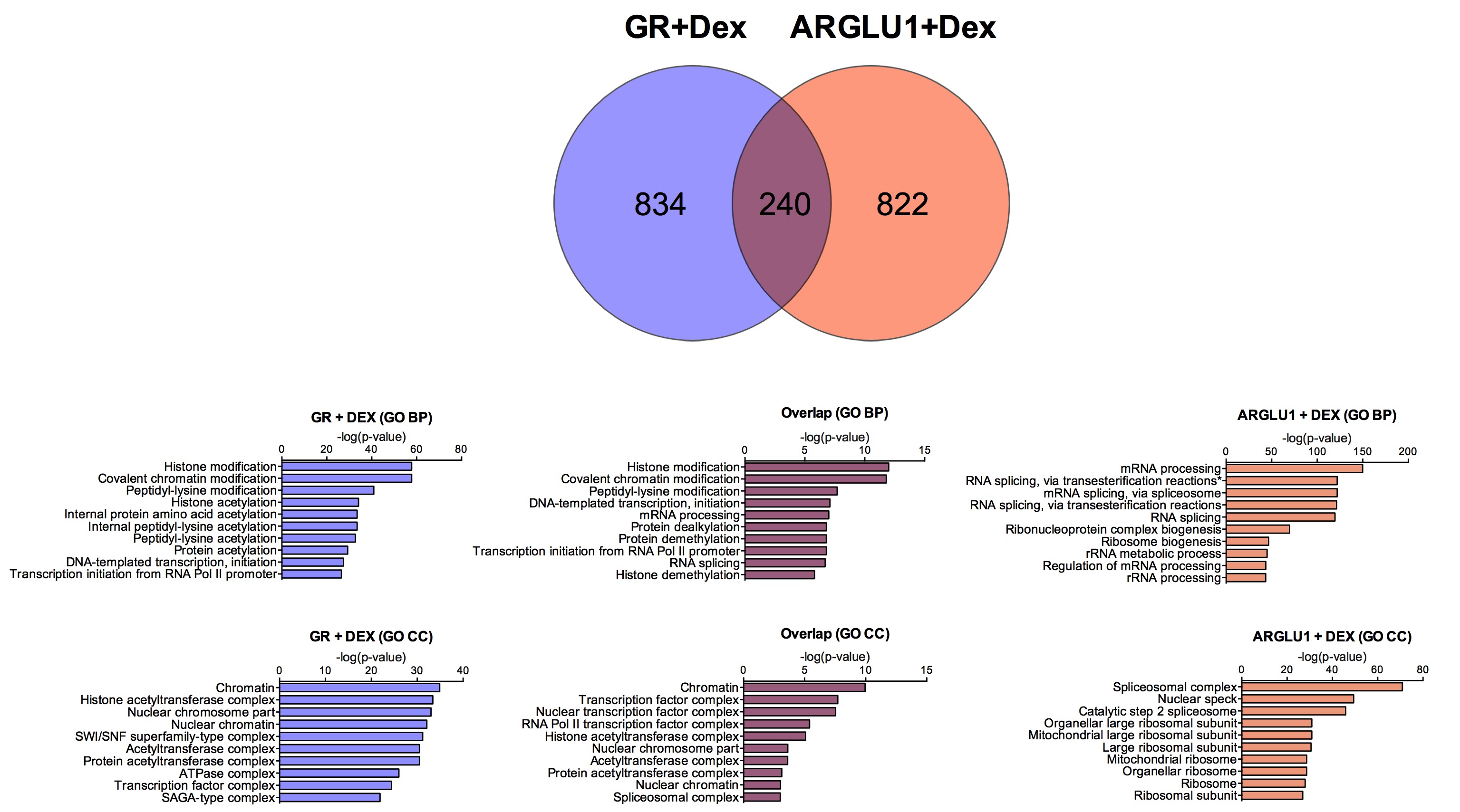
**

**Figure S8: Gene ontology enrichment of BioID-derived GR and ARGLU1 interactomes, related to Figure 3.** Streptavidin pull-downs of Dex-treated Flp-In™ T-REx™ 293 cell lines stably expressing either BirA*-GR, BirA*-ARGLU1 or BirA* identified 3415 unique proteins. 1074 proteins were over 2-fold enriched in the BirA*-GR + Dex group and 1062 proteins were over 2-fold enriched in the BirA*-ARGLU1+ Dex group compared to BirA* alone. 240 Proteins were found to be at least 2-fold enriched in both conditions. The three lists were submitted to g:Profiler to test for Gene Ontology enrichment (BP – Biological process and CC – Cellular Compartment). The conditions were set such that the minimum overlap is 2 proteins and the size of the functional category is between 3 to 500 proteins. The statistical threshold was calculated using the Benjamini-Hochberg FDR method. The top 10 most significant GO BP and GO CC terms were represented using the negative logarithm of their p-value.


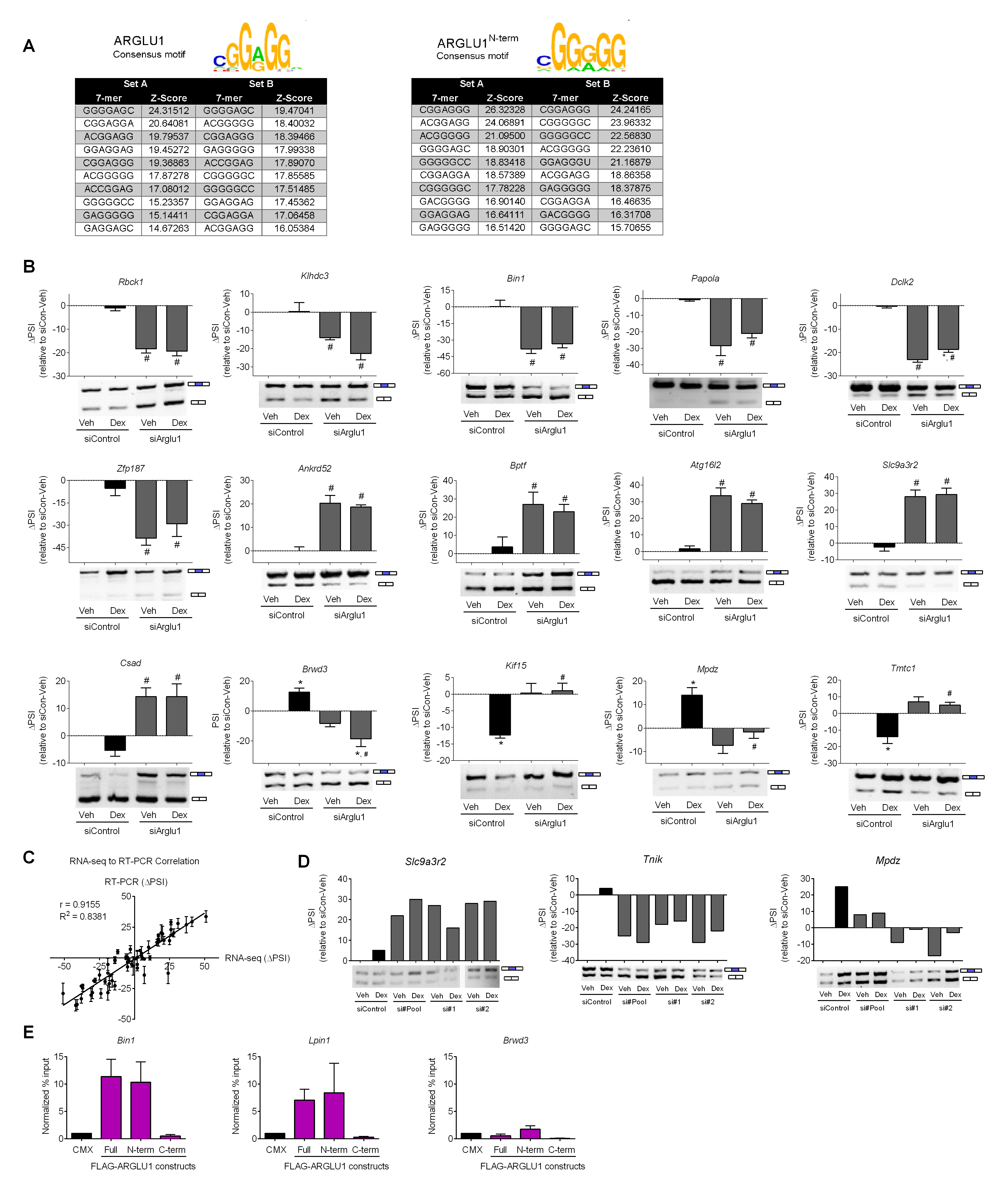


**Figure S9: ARGLU1 knockdown leads to exon skipping and exon inclusion, related to Figure 3.**

(**A)** The top ten 7-mers bound by the various ARGLU1 proteins (and corresponding Z-scores) identified by RNAcompete of GST-ARGLU1 and GST-ARGLU1^N-term^ (N-terminus intact) from Figure 3. (**B**) Splicing events with the PSI of ≥15 by RNA-seq were validated using one-step RT-PCR. Image J was used for quantification. PSI was calculated as: spliced in / (spliced in + spliced out) * 100%. ΔPSI is calculated by subtracting the individual PSIs from the PSI of siControl Veh group. Representative image is shown below the Image J quantification of the blot. Data represent the mean ± SEM (n=3). *p<0.05 vs respective Veh, #p<0.05 vs respective siControl; ANOVA followed by Neuman-Keuls test. (**C**) Correlation of ∆PSI values between RNA-seq and RT-PCR. (**D**) Changes in AS were confirmed with independent ARGLU1 siRNAs. (**E**) RNA immunoprecipitation of N2a cells transfected with full length FLAG-ARGLU1 or the indicated truncation mutants. RT-qPCR was performed on genes containing one or more putative ARGLU1 binding sites identified by visual examination within ± 300 bp of the alternatively spliced exon (*Bin1, Lpin1*) or a negative control gene that undergoes splicing but is not dependent on ARGLU1 (*Brwd3*). RNA was purified and quantified using One-Step RT-qPCR (Qiagen) with primers spanning exon-intron junctions to examine pre-mRNA binding by ARGLU1. Signals were corrected for input and expressed as normalized fold enrichment over CMX-transfected cells and are pooled from three independent experiments.

.


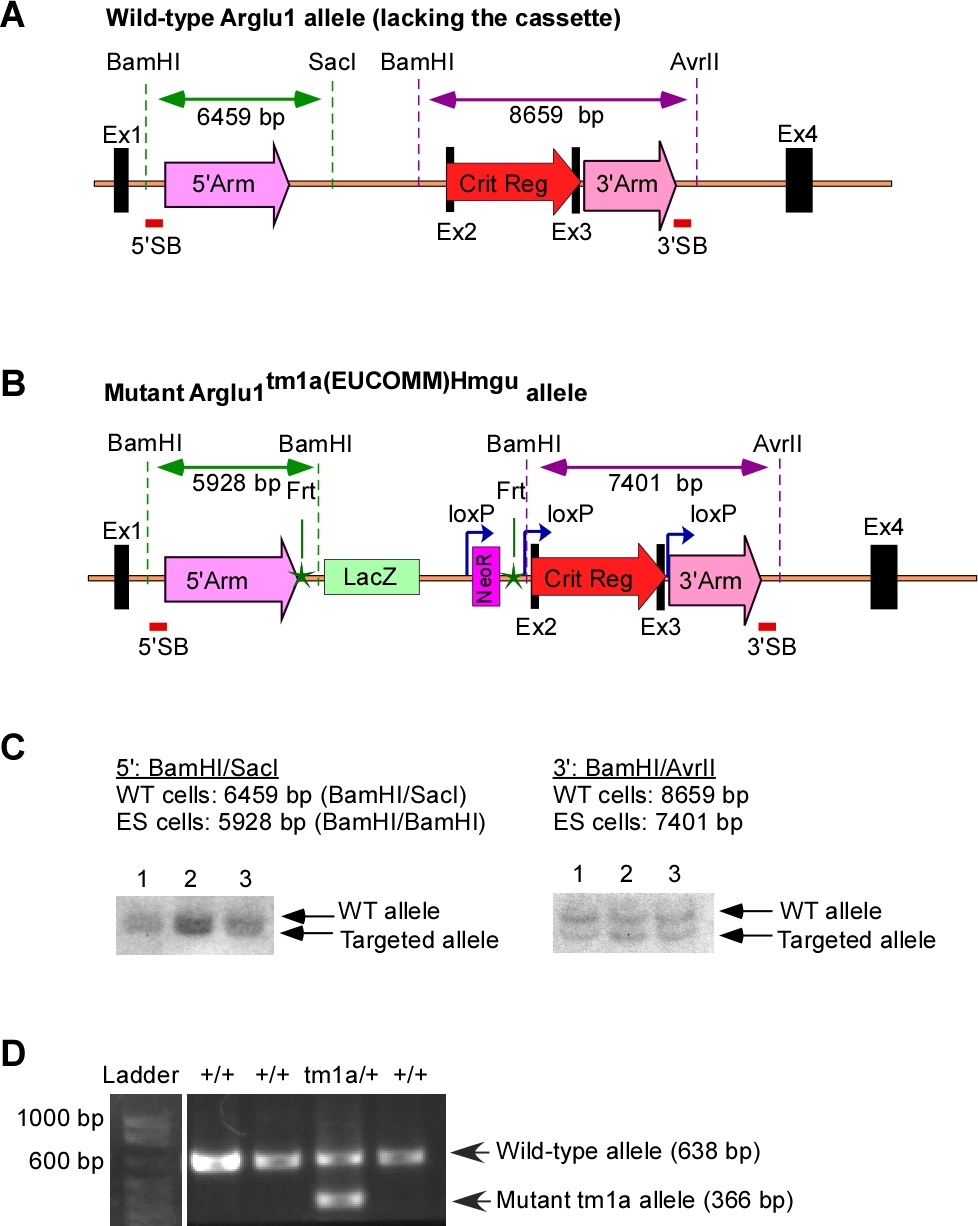


Figure S10: ES cell targeting construct and confirmation of germline transmission of ARGLU1^tm1a(EUCOMM)Hmgu^, related to Figure 5.

Southern blot was used to confirm proper cassette targeting of the 3 different ES clones purchased from EUCOMM. (**A-B**) Schematic representation of the WT (**A**) and mutant (**B**) alleles. (**C**) Southern blot of digested ES clones. Critical region between exon 2 and 3 is targeted for deletion following Cre-recombinase generating Arglu1 null allele. All three clones confirmed proper cassette targeting. 5’ and 3’ Southern blot probes (5’SB and 3’SB, respectively) were generated using primers listed in extended experimental methods. To confirm the germ-line transmission (GLT) of the knockout first Arglu1^tm1a^ allele, male founder animals derived from the ES morula aggregation were crossed to albino female mice. After crossing to albino female mice, white pups were terminated at birth, non-white pups with black eyes were genotyped for tm1a germline transmission (**D**).


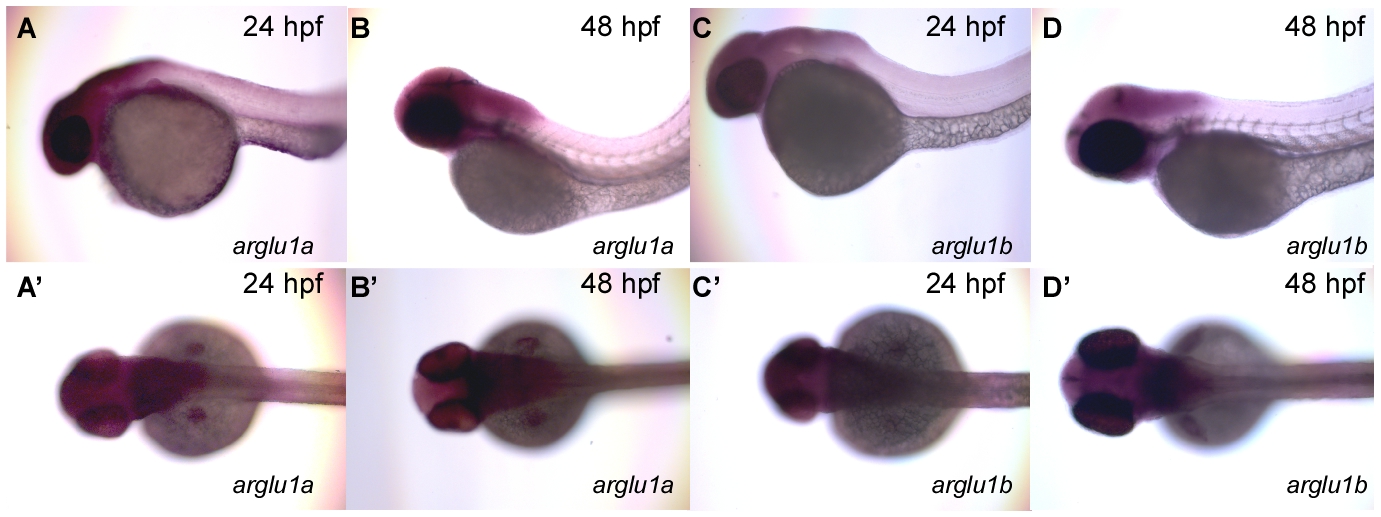


Figure S11: Expression of *arglu1a* and *arglu1b* in zebrafish, related to Figure 5.

(**A–D**) RNA *in situ* hybridization assays monitoring *arglu1a* and *arglu1b* expression in the developing zebrafish embryo. (**A, C** and **A’, C’**) expression at 24 hpf of *arglu1a* and *arglu1b*, lateral and dorsal views, respectively. (**B, D** and **B’, D’**) *arglu1a* and *arglu1b* expression at 48 hpf, lateral and dorsal views, respectively. Hpf: hours post fertilization.
